# Supplementary material for: Prevalence of hepatitis B virus infection and associated risk factors among pregnant women attending antenatal clinic in Mulago Hospital, Uganda: a cross-sectional study
Source: BMJ Open. 2020 Jun 3;10(6):e033043. doi: 10.1136/bmjopen-2019-033043 (PMC7279652; doi:10.1136/bmjopen-2019-033043)
Supplement: Author's manuscript [file bmjopen-2019-033043.draft_revisions.pdf]

BMJ Open

BMJ Open is committed to open peer review. As part of this commitment we make the peer review history of every article we publish publicly available.

When an article is published we post the peer reviewers' comments and the authors' responses online. We also post the versions of the paper that were used during peer review. These are the versions that the peer review comments apply to.

The versions of the paper that follow are the versions that were submitted during the peer review process. They are not the versions of record or the final published versions. They should not be cited or distributed as the published version of this manuscript.

BMJ Open is an open access journal and the full, final, typeset and author-corrected version of record of the manuscript is available on our site with no access controls, subscription charges or pay-per-view fees (http://bmjopen.bmj.com).

If you have any questions on BMJ Open's open peer review process please email info.bmjopen@bmj.com

BMJ Open

Hepatitis B Virus infection in pregnancy, a problem persists: A cross-sectional study among pregnant women attending antenatal care clinic in Mulago hospital, Uganda.

Journal:	*BMJ Open*
Manuscript ID	bmjopen-2019-033043
Article Type:	Original research
Date Submitted by the Author:	27-Jan-2020
Complete List of Authors:	Kayondo, Simon Peter; Makerere University College of Health Sciences, Obstetrics and Gynaecology; Association of Obstetricians and Gynaecologists of Uganda,<br>Byamugisha, Josaphat; Makere University/Mulago National Referral Hospital, Obstetrics and Gynecology<br>Ntuyo, Peter; Makere University/Mulago National Referral Hospital, Obstetrics and Gynecology
Keywords:	Hepatitis B, prevalence, pregnant women, Mulago hospital, Uganda

SCHOLARONE™
Manuscripts

I, the Submitting Author has the right to grant and does grant on behalf of all authors of the Work (as defined in the below author licence), an exclusive licence and/or a non-exclusive licence for contributions from authors who are: i) UK Crown employees; ii) where BMJ has agreed a CC-BY licence shall apply, and/or iii) in accordance with the terms applicable for US Federal Government officers or employees acting as part of their official duties; on a worldwide, perpetual, irrevocable, royalty-free basis to BMJ Publishing Group Ltd ("BMJ") its licensees and where the relevant Journal is co-owned by BMJ to the co-owners of the Journal, to publish the Work in this journal and any other BMJ products and to exploit all rights, as set out in our licence.

The Submitting Author accepts and understands that any supply made under these terms is made by BMJ to the Submitting Author unless you are acting as an employee on behalf of your employer or a postgraduate student of an affiliated institution which is paying any applicable article publishing charge ("APC") for Open Access articles. Where the Submitting Author wishes to make the Work available on an Open Access basis (and intends to pay the relevant APC), the terms of reuse of such Open Access shall be governed by a Creative Commons licence – details of these licences and which Creative Commons licence will apply to this Work are set out in our licence referred to above.

Other than as permitted in any relevant BMJ Author's Self Archiving Policies, I confirm this Work has not been accepted for publication elsewhere, is not being considered for publication elsewhere and does not duplicate material already published. I confirm all authors consent to publication of this Work and authorise the granting of this licence.

For peer review only

Hepatitis B Virus infection in pregnancy, a problem persists: A cross-sectional study among pregnant women attending antenatal care clinic in Mulago hospital, Uganda.

S.P. Kayondo[1], J. Byamugisha[2], P. Ntuyo[3].

Authors:

1. **Dr. Kayondo Simon Peter, MBChB, M.Med Obs/Gyn (MUK)**
   Project Coordinator,
   Association of Obstetricians & Gynaecologists of Uganda (AOGU)
   P.O. Box 11966,
   Kampala- Uganda.
   Email:**kayondosimondr@gmail.com**
   Mobile: +256782736078

2. **Assoc. Prof. Josaphat Byamugisha, MBChB, M.Med Obs/Gyn, Ph.D. (MUK)**
   Associate Professor,
   Department of Obstetrics and Gynaecology,
   Makerere University College of Health Sciences,
   P.O. Box 7072**,**
   Kampala- Uganda**.**
   Email: **byamugisha2001@yahoo.com**
   Mobile: +256772580330

3. **Dr. Peter Ntuyo, MBChB, M.Med Obs/Gyn (MUK)**
   Obstetrician and Gynecologist,
   Directorate of Obstetrics and Gynecology,
   Mulago Hospital,
   P.O. Box 7051**,**
   Kampala- Uganda**.**
   Email: **peterntuyo@yahoo.com**
   Mobile: +256772481636.

Correspondence author:

Dr. Kayondo Simon Peter, MBChB, M.Med Obs/Gyn (MUK)
Project Coordinator,
Association of Obstetricians & Gynaecologists of Uganda (AOGU)
P.O. Box 11966**,**
Kampala- Uganda**.**
Email:**kayondosimondr@gmail.com**
Mobile: +256782736078

For peer review only

ABSTRACT

Objective To determine the prevalence and factors associated with Hepatitis B Virus infection, among pregnant women attending antenatal care clinic in Mulago Hospital.

Design Cross-sectional observational study.

Setting Mulago National Referral Hospital, Uganda, Antenatal Care Clinic.

Participants 340 pregnant women attending their first antenatal care visit at Mulago Hospital Antenatal care clinic, were randomly selected into the study.

Primary Outcome Hepatitis B surface antigen (HBsAg) positivity.

Results A total of 340 participants were recruited, with a mean age of 27±5.7 years, and a median gravidity of 3. The prevalence of Hepatitis B Virus infection among pregnant women attending the antenatal care clinic in Mulago hospital, was found to be 2.9% (N=10). (95%CI, 1.58-5.40). The factors that were found associated with Hepatitis B virus infection were, marital status, (aOR=11.37, p=0.002), history of having had a Hepatitis B positive patient at home, (aOR=49.52, p<0.001), and history of having had a blood or body fluid splash to mucous membranes from a Hepatitis B positive patient, (aOR=61.69, p=0.015). Other factors such as age, socioeconomic status, number of sexual partners, HIV serostatus, piercing of ears, and history of blood transfusion, were not found to be significantly associated with Hepatitis B virus infection in this study.

Conclusion The prevalence of Hepatitis B virus infection among pregnant women attending antenatal care clinic in Mulago hospital, was of intermediate endemicity. The factors associated with Hepatitis B virus infection in this study population were marital status, history of having a Hepatitis B positive patient at home, and history of a blood or body fluid splash to mucous membranes from a Hepatitis B positive patient. Factors such as age, HIV status, history of blood transfusion, piercing of ears, and social status were of limited value in this study.

Key words: Hepatitis B, prevalence, pregnant women, Mulago hospital, Uganda

For peer review only - http://bmjopen.bmj.com/site/about/guidelines.xhtml

Strengths and limitations of this study

- The major strength of this study was in the diagnostic methods used. The rapid diagnostic HBsAg test, FaStep, is highly sensitive and specific, with a high accuracy, and thus results are easily reproducible. The laboratory personnel that worked on the study were highly qualified. At the Kawempe Hospital Laboratory, a senior Laboratory Technologist with extensive research experience with MUJHU, NTLP and the British Medical Research Council, and the Laboratory that carried out the follow up tests, (MBN Clinical Laboratories), such as external quality assurance HBsAg tests, HBeAg and LFTs is highly rated, and ISO 9001 certified.

- The limitations; being a crossectional study, it is a time point measure, and thus may not be able to conclude on the fact that 2.9% is the all-time prevalence. The problem may be worse.

- Secondly, the study could not help us determine if the associated factors had a causal relationship with the Hepatitis B virus infection, again, being a crossectional study.

- Finally, the study was also relatively underpowered, as the numbers found positive were small, confidence intervals wide, giving results, especially associated factors, limited precision.

Funding statement This work was supported through the DELTAS Africa Initiative grant #DEL-15-011 to THRiVE-2. The DELTAS Africa Initiative is an independent funding scheme of the African Academy of Sciences (AAS)'s Alliance for Accelerating Excellence in Science in Africa(AESA) and supported by the New Partnership for Africa's Development Planning and Coordinating Agency(NEPAD Agency) with funding from the Wellcome Trust grant #107742/Z/15/Z and the UK government. The views expressed in this publication are those of the authors and not necessarily those of AAS, NEPAD Agency, Wellcome Trust, or the UK government.

Conflict of interest The authors declare no conflict of interest.

For peer review only - http://bmjopen.bmj.com/site/about/guidelines.xhtml

Introduction.

Hepatitis B is a viral infection of the liver that can cause acute and chronic disease. Worldwide, about two billion people are exposed to Hepatitis B virus, and about 257 million people are living with Hepatitis B virus infection (defined as HBsAg positive)(1). Hepatitis B virus infection resulted in 887,000 deaths, in 2015, mostly from liver complications; liver cirrhosis and Hepatocellular Carcinoma. Hepatitis B prevalence is highest in the WHO Western Pacific Region, and the WHO African region, standing at 6.2% and 6.1% of the adult population, respectively. HBV infection and its sequelae are very common in Sub-Saharan Africa. 5-20%, is the overall HBsAg carrier rate in the general population, and is among the highest in the World. Perinatal transmission is estimated at 1-5%(2). In Uganda, 72% is the lifetime exposure. The national prevalence averages at about 10%, with regional variation, the highest prevalence being in the northern part of the country, ranging from 19% in the North West to 25% in the North East(3, 4). In pregnant women, the prevalence statistics worldwide are missing, however, in Northern Uganda, prevalence was found to be about 11.8% (5), while it was estimated at 0.9%, at Mulago Hospital, in 2016(6).

The commonest mode of transmission of Hepatitis B in highly endemic areas, is from mother to child at birth, (perinatal transmission, or vertical transmission), or through exposure to infected blood (horizontal transmission), mostly from an infected child to an uninfected child within the first five years of life(1, 7). Infants infected from their mothers, or before the age of five, very often develop chronic infection (1, 8, 9). The transmission of Hepatitis B can also be through percutaneous or mucosal exposure to infected blood and various body fluids, such as saliva, menstrual, vaginal and seminal fluids(1, 10). Spread can also happen in men who have sex with men, through reuse of injections and in injectable drug users. The infection can also be transmitted through unsafe medical, dental, and surgical procedures, as well as through practices such as tattooing, or use of sharp instruments contaminated with blood(1).

The risk of developing chronic Hepatitis B for babies born to a mother with Hepatitis B is greater than 90%, if they are not properly treated at birth. It is therefore imperative for pregnant women to know their Hepatitis B status. They should be tested early, in the first trimester(1). If tested positive, the baby, at birth, should receive both the first dose of the Hepatitis B vaccine, and the

For peer review only - http://bmjopen.bmj.com/site/about/guidelines.xhtml

Hepatitis B Immunoglobulin (a recommendation of CDC), although it may not be available in all countries (1, 11, 12). If the two are given within the first 12 hours of life, the new-born has more than 90% chance of being protected against a lifelong Hepatitis B infection (11-13).

Acute or chronic infection in pregnancy, in most cases, is similar to that in the general population, it neither increases mortality, nor yields teratogenic effects (14, 15). But with acute infection, there is a higher incidence of low birth weight and prematurity, than in the general population(15). In pregnancy, as a result of the shift in the Th1 to Th2 response, there is a depressed immune response against HBV. Hepatitis B Virus infection in new-borns is defined as HBsAg positivity 6 months after birth(1, 16). The risk of mother to child transmission is very high, in the absence of prophylaxis, varying with the HBeAg/anti-HBe status of the mother, 70-90% for HBeAg positive, 25% for HBeAg negative/HBeAb-negative mothers and 12% for HBeAg-negative/anti HBe positive mothers (13, 15, 17).

Study objectives

General objective

To determine the prevalence and factors associated with Hepatitis B Virus infection among pregnant women attending antenatal care clinic in Mulago Hospital.

Specific objective

1. To determine the prevalence of HBV infection among pregnant women, attending ANC clinic in Mulago Hospital.
2. To establish the factors associated with HBV infection, among pregnant women attending ANC clinic, in Mulago Hospital.

For peer review only - http://bmjopen.bmj.com/site/about/guidelines.xhtml

Methods:

Study design and setting

This was a cross sectional study, conducted at Mulago Hospital antenatal care clinic. Mulago hospital, is located in Kampala City, the capital of Uganda. It is a public, general and teaching hospital, with a bed capacity of more than 1500 beds, founded in 1917. It is located on Mulago hill, approximately 5km Northeast of Kampala's central business district. The hospital's ANC unit attends to approximately 2500 patients per month, making a wide sampling population for the researcher.

Mulago Hospital was purposively selected because, it is a National Referral Hospital for Uganda, that serves people from all ethnic groups, races, and regions of Uganda, as well as refugees, such as those from Rwanda, South Sudan and Congo. Uganda currently has a population of more than 34 million people. It is also the hospital where the researcher was based, as a Master student, making the conduction of the study pretty convenient for him.

The Mulago Hospital ANC Clinic, is housed at Kawempe General Hospital, in Kawempe Division, temporarily, as the main Mulago Hospital is undergoing major renovation. The clinic continues to offer preventive and curative services, such as health education, immunization, prenatal maternal and fetal monitoring, among others. The clinic serves between 200 and 250 ANC mothers per day, of whom about 100 are attending their first ANC visit. It runs three days a week, Tuesday, Wednesday and Thursday, and first visit attendees are served on each of these days. Only mothers attending their first visit were enrolled into the study, as the flow of first visit attendees in the ANC clinic was convenient for recruitment, unlike their repeat visit counterparts, as it was done right after their health education session, as they awaited examination

Study population

The study population was, pregnant women attending antenatal care clinic, between December 2018 and February 2019, in Mulago Hospital, Kawempe site.

Eligibility criteria

Inclusion criteria:

All pregnant women who were attending their first ANC visit at Mulago ANC clinic, and were willing to participate in the study. All women above the age of 18 years were enrolled, as well as those below this age, who were considered as emancipated minors.

For peer review only - http://bmjopen.bmj.com/site/about/guidelines.xhtml

Exclusion criteria:

Pregnant women who could not understand either English or Luganda. This is because these were the two languages used for the consenting process, and administration of the questionnaires.

Research variables:

The dependent variable was HBV infection among pregnant women. (HBsAg positivity)

The independent variables were the factors that are thought to be associated with the positive HBV status. These include the socio-demographic factors such as age, marital status, level of education, socio economic status and sexual behavior. Others are Obstetric factors, such as gravidity and parity. Other factors are; history of blood transfusion, ear piercings, tattoos, injection drug use, history of caring for a HBV positive patient, immunization status, among others.

Sample size estimation

Sample size for prevalence, was estimated using the Kish and Leslie formula, 1965 (18).

Using a prevalence from Northern Uganda of 11.8% (5)

The sample size for prevalence, would be 160 women.

The sample size estimation for factors associated with Hepatitis B Virus infection in pregnancy, was based on the hypothesis that the prevalence of HBV would be higher among women who are below 20 years than among those who are above 20 years (Comparing HBV infection among younger women, versus older women), as found by Bayo et al(5). The formula for comparing two proportions by Fleiss was used (REF: Fleiss, Statistical Methods for Rates and Proportions, formulas pages 3.18 & 3.19) and as applied in the OpenEpi Calculator accessed at:

http://www.openepi.com/SampleSize/SSCC.htm

Assuming power of 80%, type 1 error of 5% and the frequency of HBV of 20% among women 20 years and below and frequency of 8.7% among those over 20 years, based on a cross sectional study by Bayo P et al, in Lacor and Gulu Hospitals, Northern Uganda(5)

Total sample size for associated factors was 340

This sample size, 340, was larger than the initial 160 for prevalence estimate, and therefore, sample size, **340 pregnant women**, was used.

For peer review only - http://bmjopen.bmj.com/site/about/guidelines.xhtml

Sampling procedure

The sampling method that was used, is a systematic random probability sampling method, where by participants were obtained during the day of data collection, after obtaining the register book for the antenatal care clinic attendance, and the booking cards for the first visit attendees. Only pregnant women attending their first antenatal visit at Mulago antenatal care clinic were enrolled. The booking cards for the first visit attendees were collected as they came in to the clinic, on the day of data collection, and every 10th card owner was requested to participate in the study. With an average daily attendance of about 100 first ANC visit pregnant women, and the ANC clinic running three days a week, 12 days in 4 weeks, to collect data over a period of three months, and complete the sample size of 340 subjects, every 10th woman was enrolled into the study. (100/10= 10 participants/day, 10X12X3=360 participants in three months). If the 10th woman declined participation in the study, or was ineligible, then the 11th was considered.

Study Procedure

Data was collected between December 2018 and February 2019.

Pregnant women who had come to the antenatal clinic for their first visit, made the sampling frame. This was because of the ease of recruitment after counselling. Systematic random sampling technique, was used to arrive at the sampling units. The booking cards for the first ANC visit attendees were collected as they came in to the clinic on the day of data collection, and every 10th card owner was asked to participate in the study. Participants were approached by the antenatal clinic midwives, who were the research assistants, and the study purpose explained to them. They were then screened for eligibility, and asked if they were willing to participate in the study. Then informed consent was obtained.

Interviewer-administered semi-structured questionnaires, were used to obtain socio-demographic information such as; age, gestation age, gravidity, parity, education and occupation, as well as other factors that could influence infection with HBV, such as injection drug use, tattoos, family history of HBV infection, sexual history, among others. Obstetric factors like gravidity and parity, previous history of taking care of a HBV positive patient, immunization against HBV, were all assessed.

For peer review only - http://bmjopen.bmj.com/site/about/guidelines.xhtml

Four millilitres (4mls) of whole blood were collected aseptically into BD Clot Activator Tube vacutainers from the antecubital fossa, using WHO (2010) guidelines on blood collection(19). The blood was then taken to the Kawempe Hospital Laboratory, centrifuged, and an aliquot of serum, about 2mls, was transferred into cryovials. Immunochromatographic assay using HBsAg Rapid Test Strip, FaStep, was used to do HBsAg test on the samples following the manufacturer's instructions (Appendix V). This rapid test kit has a relative sensitivity of >99.9%, a relative specificity of 99.9%, and an accuracy of >99.9%. The HBsAg rapid tests were carried out by the laboratory assistant, using the following procedure: A rapid test strip was labelled with the patient identifiers, and placed on a flat surface. 3 drops of serum was pipetted from the cryovial, and applied to the sample pad of the test strip. The result was read after 15 minutes. A positive result was one where two coloured bands appeared on the membrane, one in the control region, and another in the test region. A negative result was one, where only one band appeared, in the control region, and none in the test region. An invalid result, was one where a band failed to appear in the control region.

Results were produced and a copy given to the patient after being counseled by the principal investigator, and the nurse counsellor. All HBsAg positive samples were sent to MBN Clinical Laboratories, Kampala, an ISO accredited laboratory, for HBsAg retesting for confirmation, HBeAg testing, and liver function tests. 5% of all HBsAg negative samples were also taken to MBN, for external quality assurance. All samples were packed and transported at 2-8 degrees celcius, on the same day of collection. HBV positive mothers were referred to the Hepatitis B Clinic in Mulago Hospital (Kiruddu) for further care, where their viral load assays would be done, and further assessed for treatment. A plan to vaccinate their babies within 24 hours after birth with the Hepatitis B Vaccine was also made, and coordinated through the Mulago Hospital ANC clinic. (The study endeavoured to meet the cost of the HepB-BD vaccine, for the positive mothers).

Data Management and Analysis

Double raw data entry into Epidata 3.1, was done by the principal investigator, and imported into STATA 14.1

Descriptive statistics such as mean, standard deviation and percentages were determined. Results are presented numerically as frequencies, percentages, in charts and tables. HBV prevalence was

For peer review only - http://bmjopen.bmj.com/site/about/guidelines.xhtml

determined, that is the number of HBsAg positive pregnant women, divided by the number of all women recruited into the study. To assess factors associated with Hepatitis B virus infection in pregnancy, bivariate analysis was performed, to compute crude odds ratios at the 95% level of significance. At bivariate analysis, the t test was used to analyse continuous variables such as age, while the Fischer's exact test was used to analyse categorical variables.

To assess independent association of these risk factors, multivariate analysis was performed, where all independent variables with a p-value of less than 0.2 at bivariate analysis, and those with biological significance, were considered for multivariate logistic regression. At multivariate analysis, stepwise regression method was used to arrive at the model of best fit. Associations with p-value as adjusted Odds Ratio of 95% Confidence Interval, were considered statistically significant. The results of the analysis are presented in tables.

Ethical considerations

Permission to carry out the study was obtained from the Department of Obstetrics and Gynaecology, Makerere University, and Makerere University School of Medicine Research and Ethics Committee, **(#REC REF 2018-119),** Mulago Hospital Research and Ethics Committee, **(#MHREC1519**), as well as the Uganda National Council for Science and Technology, **(#HS257ES),** before any collection of samples and data.

Information regarding the study was explained to the subjects in English/Luganda and written consent for participation in the study was obtained in English/Luganda. Interviews were conducted in privacy of the participant, and samples were collected following local guidelines, in privacy of the participant. Only study numbers with unique participant identifiers, were used to label the questionnaires and the samples, and no names were used on questionnaires or samples. Filled questionnaires were kept separate from signed consent forms, and locked away securely with only access to study team. Mothers had an informed consent taken, after the study aim, procedures risks and benefits were explained, in a language they understood well. They could also choose to drop out at any point of this study, with an assurance that if they did so, they would not be denied

For peer review only - http://bmjopen.bmj.com/site/about/guidelines.xhtml

any other ANC services. Mothers below the age of 18 were allowed to consent, as these are emancipated minors.

Only Hepatitis B negative health workers, who were fully immunized against Hepatitis B, were enrolled as research assistants. And they wore protective gear, such as gloves, masks, and eye glasses during laboratory procedures. These measures were to protect both mothers and health workers from acquiring Hepatitis B virus infection from each other during the study.

Patient and Public involvement While there were patients involved in this study as study participants, they were not involved at the stage of the study design. But the intended outcome in favour of the patient was that Hepatitis B birth dose vaccination would be made available for all exposed babies, which is in the patients' interest. A summary of the findings will be circulated using sms messaging to the phone contacts of all study participants.

Results.

Description of the study population

A total of 340 participants were recruited into the study, with a mean age of 27±5.7 years. More than half, (56.8%), were from Kampala, 40% from Wakiso, and 3.2% from elsewhere**.** The majority of the participants, (76.7%) had a secondary education and above, and 52.4% were employed. A little over a half of the participants, (51.7%), were business people, 41.5% in professional jobs, 5.1% casual laborers, while 3% were health workers. More than half of them (65.3%), were of low socioeconomic status, earning less than sh. 500,000 per month. Majority of the participants (70.3%), had had their sexual debut during their teenage years.

For peer review only - http://bmjopen.bmj.com/site/about/guidelines.xhtml

Table 1: Table showing demographic/gynecological/obstetric/sexual characteristics of the study participants

Demographic Characteristics	Freq [N=340, (%)]
**Age (years) ; mean (std.dev)**	27 (5.7)
**Age (years) categorized**	
≤20	28 (8.2)
>20	312 (91.8)
**Employment status**	
Yes	178 (52.4)
No	162 (47.6)
**Marital status**	
Staying with partner	304 (89.4)
No stable partner	36 (10.6)
**Gyn/Obs/Sexual characeristics**	
**Gravidity; median (IQR)**	3 (2 - 3)
**Gravidity categorized**	
1	67 (19.7)
2-4	211 (62.1)
>4	62 (18.2)
**Weeks of amenorrhea (WOA) categorized**	
>20	211 (62.1)
≤20	54 (15.9)
Unknown	75 (22.1)
**Mother Hepatitis B positive?**	
Yes	4 (1.2)
No	126 (37.1)
I don't know	210 (61.7)
**Number of sex partners; median (IQR)**	2 (1-3)
**Number of sex partners categorized**	
1	117 (34.6)
≥2	221 (65.4)

For peer review only - http://bmjopen.bmj.com/site/about/guidelines.xhtml

Factors associated with Hepatitis B virus infection

Table 2: Table showing Immunosuppression characteristics, vaccination history, familial exposure, occupational exposure risk of study participants, and exposure to Hepatitis B through sharp instruments, Medical and Surgical Procedures.

Immunosuppression characteristics	Freq [N=340, (%)]
**HIV sero-status**	
Positive	10 (2.9)
**History of Diabetes Mellitus**	
Yes	3 (0.9)
No	47 (13.8)
Unknown	290 (85.3)
**Vaccination history**	
**Hepatitis B vaccination in first year of life**	
Yes	9 (2.6)
No	240 (70.6)
Don't know	91 (26.8)
**Hepatitis B vaccination after the age of 10 years**	
Yes	12 (3.5)
No	299 (88.0)
Don't know	29 (8.5)
**Exposure through sharp instruments/medical/surgical procedures**	
**Ever had blood transfusion**	
Yes	18 (5.3)
**Ever pierced your ears**	
Yes	275 (80.9)
**Familial exposure**	
**Have a Hepatitis B positive patient at home**	
Yes	7 (2.1)
No	291 (85.6)
Don't know	42 (12.3)
**Occupational exposure**	
**Ever had needle stick injury**	
Yes	4 (1.2)
**Blood or body fluid splash from Hepatitis B positive patient.**	
Yes	2 (0.6)

Only 2.9% of study participants were HIV positive. Vaccination against Hepatitis B was low, with 2.6%, and 3.5% as the childhood and adult vaccination rates, respectively. About 2.1% of the participants reported having a Hepatitis B positive patient at home.

For peer review only - http://bmjopen.bmj.com/site/about/guidelines.xhtml

Prevalence

10 out of 340 participants were HBsAg positive, comprising 2.9%. (95%CI, 1.58-5.40)

All the HBsAg positive participants were HBeAg negative, and had normal liver function tests.

Associations between the independent variables and Hepatitis B status.

Table 3: Table showing association between demographic, gynecological, obstetric characteristics, and Hepatitis B status

**Demographic characteristics**	**Hepatitis B status**		**Unadj Odds (95% CI)**	**P-value**
	**Pos [n=10, (%)]**	**Neg [n=330, (%)]**		
**Age(years); mean (std.dev)**	27.4 (6.2)	26.9 (5.7)		**0.775[a]**
**Age (years) categorised**				**0.582**
<20	1 (10.0)	27 (8.2)	1.00	
≥20	9 (90.0)	303 (91.8)	1.25 (0.15 - 10.21)	
**Employment status**				**0.753**
Yes	6(60)	172(52.2)	1.00	
No	4(40)	158(47.8)	0.73(0.20-2.62)	
**Marital status**				**0.004**
Staying with partner	6 (60.0)	298 (90.3)	1.00	**0.014**
No stable partner	4 (40.0)	32 (9.7)	6.21 (1.66 - 23.16)	
**Gynecological/Obstetric factors**				
**WOA categorized**				**<0.001**
>20	1 (10.0)	210 (63.6)	1.00	
≤20	6 (60.0)	48 (14.6)	26.25 (3.09 - 223.13)	
Unknown	3 (30.0)	72 (21.8)	8.75 (0.90 - 85.46)	
**Mother Hepatitis B positive?**				**0.036**
Yes	1 (10.0)	3 (0.9)	1.00	
No	1 (10.0)	125 (37.9)	0.02 (0.00 - 0.48)	
I don't know	8 (80.0)	202 (61.2)	0.12 (0.01 - 1.27)	

P- Fishers exact p-values, P[a]- t test p-values

At bivariate analysis, the participant's marital status, weeks of amenorrhea, mother's Hepatitis B status were found significantly associated with Hepatitis B status, and were taken to multivariate analysis, along with age, for biological significance.

For peer review only - http://bmjopen.bmj.com/site/about/guidelines.xhtml

Table 4: Association of Immunosuppression characteristics/occupational exposure risk/surgical procedures/sharp instruments and familial exposure with Hepatitis B status.

Immunosuppression characteristics	Hepatitis B Status		Unadjusted Odds (95% CI)	P-value
	Pos [n=10, (%)]	Neg [n=330, (%)]		
**HIV sero-status**				**0.261**
Positive	1 (10.0)	9 (2.7)	3.96 (0.45 – 34.69)	
Negative	9 (90.0)	321 (97.3)	1.00	
**Occupational exposure risk**				
**Blood or body fluid splash from a Hepatitis B positive patient**				**0.058**
Yes	1(10)	1(0.3)	36.56 (2.11 – 631.85)	
No	9(90)	329(99.7)	1.00	
**Exposure through surgical procedures/sharp instruments**				
**Ever had blood transfusion**				**0.424**
Yes	1 (10.0)	17 (5.2)	2.05 (0.24 – 17.09)	
No	9 (90.0)	313 (94.8)	1.00	
**Ever pierced your ears**				**0.411**
Yes	7 (70.0)	268 (81.2)	1.00	
No	3 (30.0)	62 (18.8)	1.85 (0.47 – 7.37)	
**Familial exposure**				
**Have a Hepatitis B positive patient at home**				**0.011**
Yes	2 (20.0)	5 (1.5)	1.00	
No	6 (60.0)	285 (86.4)	19.00 (3.05 - 118.24)	
Don’t know	2 (20.0)	40 (12.1)	2.38 (0.46 – 12.17)	

Having had a blood or body fluid splash to the mucous membranes from a Hepatitis B positive patient, and having a Hepatitis B positive patient at home were found significantly associated with Hepatitis B status, at bivariate analysis. Both were taken to multivariate logistic regression analysis.

For peer review only - http://bmjopen.bmj.com/site/about/guidelines.xhtml

Table 5: **Multivariable logistic regression model showing factors associated with Hepatitis B infection among study participants**

Characteristics	Adjusted Odds ratio (95% CI)	P-value
**Age(years) categorized**		
<20	1.00	
≥20	0.27 (0.01 - 5.31)	**0.387**
**Marital status**		
Staying with partner	1.00	
No stable partner	11.37 (2.37 - 54.60)	**0.002**
**Have a Hepatitis B positive patient at home**		
No	1.00	
Yes	49.52 (6.24 - 392.85)	**<0.001**
Don't know	4.25 (0.71 - 25.35)	**0.113**
**Blood or body fluid splash from Hepatitis B positive patient**		
No	1.00	
Yes	61.69 (2.24 - 1701.54)	**0.015**

Variables from bivariate analysis with a p-value less than 0.2 were taken to multivariable logistic regression analysis. Individual logistic regressions were done on each of these, and variables with p-values less than 0.05 were adjusted into a final logistic regression model. The final model of best fit was arrived at using the stepwise regression method.

Marital status, having had a Hepatitis B positive patient at home, and history of a blood or body splash to mucous membranes from a Hepatitis B positive patient were found significantly independently associated with Hepatitis B Virus infection, with p values < 0.05. Age was not significant.

Data availability statement All data relevant to this study are included in the article or uploaded as supplementary information. Raw data accessible on: https://drive.google.com/drive/folders/1wc0C3bP4Qge0OKtG4z-zXC6pbD1F3nsL?usp=sharing

For peer review only - http://bmjopen.bmj.com/site/about/guidelines.xhtml

Discussion

This study was conducted to determine the prevalence and factors associated with Hepatitis B Virus infection, among pregnant women attending the antenatal care clinic in Mulago hospital. A total of 340 women participated in this study.

Prevalence

In this study, the prevalence of Hepatitis B Virus infection among pregnant women attending the antenatal care clinic in Mulago hospital was found to be 2.9% (N=10)(95%CI, 1.58-5.40) This prevalence, lies in the Hepatitis B intermediate endemicity WHO category (2-8%)(20). It is comparable to the one found by Pirillo et al in Kampala (Uganda), 4.9% and Kigali (Rwanda), 2.4%, in 2007 (21), and by Mutagoma et al in Rwanda, 3.7%(22). Their study populations were a bit different however, as they did their studies among HIV positive pregnant women only, whereas ours was among both HIV positive and negative pregnant women. Other studies by Ngaira J et al in Mbagathi district in Kenya, and by Chernet et al in Ethiopia, found the prevalence of Hepatitis B among pregnant women attending Antenantal care to be 3.8%, and 3.5% respectively, which are still comparable to the one found in this study, and in a similar setting(23, 24).

This prevalence is still high however, in comparison to low endemicity areas, such as the United States of America, at 0.38% found by Walker et al(25). These low endemicity areas have vigorously implemented Hepatitis B prevention strategies, such as; universal testing of all pregnant women for Hepatitis B, universal birth dose vaccination and universal childhood immunization; unlike intermediate and high endemicity areas(26). It is also higher than the one estimated by Namirembe et al, 0.9%, among pregnant women in Mulago Hospital, in early 2016, before Mulago Hospital was transferred to the new Kawempe site(6). The explanation to this difference may lie in the fact that Kawempe, the site at which this study was done, is a slum area, with many risky activities such as prostitution, increasing the risk profile of participants, as compared to the old Mulago site, where the earlier study was done. The other contributors to the difference could be in the sensitivity and specificity of the tests and testing procedure used in the two studies, the selection criteria of participants, and the spatial difference.

The 2.9% prevalence is however much lower than the 11.8% prevalence found by Bayo et al in highly endemic Northern Uganda, among pregnant women in 2014(5), and even much lower than

For peer review only - http://bmjopen.bmj.com/site/about/guidelines.xhtml

the average National prevalence of about 10% in the general population, as estimated by Bwogi et al from a National survey in 2009(4). It is still lower than the prevalence found in other select populations such as health workers in Mulago Hospital, found at 8.1% by Abdallah K Ziraba et al(27). The explanation for the difference in prevalence could be multipronged; the Central region being a low prevalence area, compared to other regions of Uganda, such as the North and East, as well as the fact that there has been an intensification of prevention programmes against Hepatitis B in the country, over the last few years, such as increased awareness, on the mode of spread and prevention. These all could have contributed to a reduction in prevalence of Hepatitis B in the general population, and hence among pregnant women.

Factors associated with Hepatitis B Virus infection among pregnant women

In this study, the factors that were found to be independently associated with Hepatitis B virus infection were; marital status, history of having had a Hepatitis B positive patient at home, and history of having had a blood or body fluid splash from a Hepatitis B positive patient. Other factors that have been found to be significant in studies elsewhere, such as age(5, 25, 28, 29), level of education,(29, 30) socioeconomic status(31), number of sexual partners(24, 32), HIV serostatus (28, 31), piercing of ears(32), history of abortions(23, 24), history of blood transfusion(28), and history of jaundice,(33), among others, were not found to be significantly associated with Hepatitis B virus infection in this study.

Marital Status

In this study, it was found that women who did not have a stable partner, were eleven (11) times as likely to be Hepatitis B positive, as their counterparts who were staying with partners. (aOR=11.37, 95% CI, (2.37-54.60), p= 0.002). An almost similar finding was noted by Kirbak et al in Juba, South Sudan, although the increased risk was specific to loss of a partner; (OR 4.4, CI 1.4 -13.9)(33). Chernet et al, and Umare et al also found an association between multiple sexual partners and Hepatitis B positivity among pregnant women in Ethiopia, (aOR=6.923) and (aOR=16.8), respectively(24, 32). Whereas this is a little different from the lack of a stable partner, but it outlines the risk associated with having no stable partner.

This finding is however contrary to findings by Ngaira et al in Kenya, where marital status was not found to be significantly associated with Hepatitis B status,(23) and by Buseri et al, in Niger

For peer review only - http://bmjopen.bmj.com/site/about/guidelines.xhtml

state, Nigeria(34). The reasoning behind this association however, could be the fact that some of the women with no stable partner were widowed, separated, or unmarried, which increases the risk of having contracted Hepatitis B from the lost partner, or which increases risky behavior, leading to higher chances of contracting the Hepatitis B virus.

History of having had a Hepatitis B positive patient at home.

Women who reported a history of having had a patient at home positive for Hepatitis B Virus, were almost fifty (50) times more likely to be positive for Hepatitis B, than those who did not.(aOR=49.52, 95% CI, (6.24-392.85), p<0.001). CDC supports this finding, indicating that 3-20% of people in contact with Chronic Hepatitis B patients have been found with evidence of Chronic Hepatitis B infection themselves, thus calling for screening and vaccination of all these contacts(35). This finding also rhymes with what Kirbak et al found in Juba, South Sudan, an increased risk of Hepatitis B infection among pregnant women who reported history of loss of a partner(OR=4.4)(33). This important finding underscores the risk of horizontal transmission of Hepatitis B virus infection, either sexually between partners, or through contact with blood and body fluids, among people staying in the same home. This can be through sharing household items such as toothbrushes, razors, syringes and needles, among other things. The risk posed by having to take care of a Hepatitis B positive patient is even higher, with exposure to blood and body fluids infected with the virus.

However, in the event that the Hepatitis B positive patient at home was the mother, the risk of vertical transmission holds as well, with chances of the participants having contracted the infection from their mothers at birth. All in all, it is essential that those who live with, or take care of a Hepatitis B positive patient be fully vaccinated against the same to reduce the risk. And all mothers should be tested during ANC and vertical transmission prevented through birth dose Hepatitis B vaccination of the exposed infant, within the first 24 hours.

History of having had a blood or body fluid splash to the mucous membranes, from a Hepatitis B positive patient.

Study participants who had ever had a blood or body fluid splash to mucous membranes from a Hepatitis B positive patient, were almost 62 times more likely to be Hepatitis B positive than those who had never had that experience (aOR=61.69, 95%CI, (2.24-1701.54), p=0.015). In a study done by Ziraba et al among health workers in Mulago Hospital, where they reported the prevalence of

For peer review only - http://bmjopen.bmj.com/site/about/guidelines.xhtml

Hepatitis B to be 8.1% in this population, it was reported that exposure to potentially infectious body fluids was high in this population, and yet a small percentage of the health workers were vaccinated(27). This therefore is still a significant route of potential infection with the Hepatitis B virus, be it from an infected patient to a health worker, or from a Hepatitis B positive patient to their caretaker, especially if the health worker or the caretaker are not vaccinated against Hepatitis B.

WHO estimates the risk of infection with Hepatitis B after a single percutaneous exposure to be 6-60%, which is higher than that of Hepatitis C, 2%, and that of HIV, 0.1-0.3% (36) It is not a surprise then, that some of the Hepatitis B positive participants in our study were health workers, and some of them admitted to this risk, while others had nursed a Hepatitis B positive patient at some point in their life, with a similar risk. Therefore the need to vaccinate especially health workers and people in contact with patients positive for Hepatitis B remains paramount(37).

Study Limitations

1. Being a crossectional study, it is a time point measure, and thus may not be able to conclude on the fact that 2.9% is the all-time prevalence. The problem may be worse.

2. The study could not help us determine if the associated factors had a causal relationship with the Hepatitis B virus infection, being a crossectional study.

3. The study was also relatively underpowered, as the numbers found positive were small, confidence intervals wide, giving results, especially associated factors, limited precision

Conclusion

The prevalence of Hepatitis B virus infection among pregnant women attending antenatal care clinic in Mulago hospital was of intermediate endemicity.

The factors associated with Hepatitis B virus infection in this study population were marital status, history of having had a Hepatitis B positive patient at home, and history of a blood or body fluid splash to mucous membranes from a Hepatitis B positive patient.

Factors such as age, HIV status, history of blood transfusion, piercing of ears, and social status were of limited value in this study.

For peer review only - http://bmjopen.bmj.com/site/about/guidelines.xhtml

Recommendations

Seeing as the prevalence found lies in the intermediate endemicity category, testing of all pregnant women for Hepatitis B virus during antenatal care is recommended. A birth dose Hepatitis B vaccine, with or without Hepatitis B Immunoglobulin for all babies born to Hepatitis B positive mothers, followed with two other doses within the first six months of life, is recommended, to curb mother to child transmission.

Since the majority of the adult population did not get childhood vaccination, which was only introduced in Uganda in 2002, screening and adult vaccination with 3 doses of Hepatitis B vaccine is recommended for high risk groups, such as health workers, people living or taking care of Hepatitis B positive patients, and women without stable partners, to control horizontal transmission.

Considering the significance of this prevalence among pregnant women, vaccination of pregnant women with the 3 doses of the Hepatitis B vaccine, within six months, is recommended, as it is not contraindicated in pregnancy, and yet protective.

A wider study, preferably multicenter, with a larger sample size, and a wider variety of tests, such as viral load, Anti-Hepatitis B Core Antibody, should be carried out in this same study population. This will ascertain the exposure status to Hepatitis B virus, the risk of vertical transmission, and more generalizable associated factors.

Authors' contributions

KSP developed the study's concept, designed the study methodology, and also participated in data collection, analysis and interpretation. PN revised the manuscript critically, and made amendments for important intellectual content; JB contributed significantly to the intellectual content of the manuscript and also gave the final approval of the version submitted. All of the authors have agreed to be accountable for all aspects of the work in this study.

For peer review only - http://bmjopen.bmj.com/site/about/guidelines.xhtml

Authors' information

KSP is an Obstetrician and Gynaecologist, Department of Obstetrics and Gynaecology, Makerere University College of Health Sciences. PN is an Obstetrician and Gynaecologist, Directorate of Obstetrics and Gynecology, Mulago Hospital. JB is an Associate Professor, Department of Obstetrics and Gynaecology, Makerere University College of Health Sciences.

Acknowledgements

We want to thank all senior members of the Department of Obstetrics and Gynecology, Makerere University and members of the Directorate of Obstetrics and Gynaecology, Mulago hospital, for the support and direction during the study. In a special way, we thank Assoc. Prof. Annettee Nakimuli, for her tremendous input to this study and providing a conducive environment for it to be carried out. Great thanks to Mr Godwin Anguzu, who offered technical input during data analysis. Finally, great thanks to the whole dedicated data collection team in the Mulago Hospital antenatal care clinic, Kawempe site.

REFERENCES

1. Organisation WH. Hepatitis B Fact Sheet World Health Organisation; 2018 [
2. Kiire C, Group ARS. Hepatitis B infection in sub-Saharan Africa. Vaccine. 1990;8:S107-S12.
3. Ochola E, Ocama P, Orach CG, Nankinga ZK, Kalyango JN, McFarland W, et al. High burden of hepatitis B infection in Northern Uganda: results of a population-based survey. BMC Public Health. 2013;13(1):727.
4. Bwogi J, Braka F, Makumbi I, Mishra V, Bakamutumaho B, Nanyunja M, et al. Hepatitis B infection is highly endemic in Uganda: findings from a national serosurvey. African health sciences. 2009;9(2).
5. Bayo P, Ochola E, Oleo C, Mwaka AD. High prevalence of hepatitis B virus infection among pregnant women attending antenatal care: a cross-sectional study in two hospitals in northern Uganda. BMJ Open. 2014;4(11):e005889.

For peer review only - http://bmjopen.bmj.com/site/about/guidelines.xhtml

For peer review only

6. Allen N, Bashir M, Mugisha TI. Prevalence and Associated Factors of Hepatitis B Virus Infection among Pregnant Women Attending Antenatal Care Clinic at Mulago National Referral Hospital, Uganda. International Blood Research & Reviews. 2017:1-10.
7. Paul T, Marie TP, Bechem E. Knowledge, attitude and practice of staff of 4 hospitals in Yaoundé on the prevention of vertical transmission of hepatitis B. Pan African Medical Journal. 2017;28(1).
8. Ifeorah I, Bakarey A, Adewumi M, Faleye T, Akere A, Omoruyi C, et al. Patterns of serologic markers of hepatitis B virus infection and the risk of transmission among pregnant women in Southwestern Nigeria. Journal of Immunoassay and Immunochemistry. 2017;38(6):639-51.
9. Shi Y-H, Shi C-H. Molecular characteristics and stages of chronic hepatitis B virus infection. World journal of gastroenterology: WJG. 2009;15(25):3099.
10. Bodo B, Malande OO. Delayed introduction of the birth dose of Hepatitis B vaccine in EPI programs in East Africa: a missed opportunity for combating vertical transmission of Hepatitis B. The Pan African Medical Journal. 2017;27(Suppl 3).
11. Prevention CfDCa. Viral hepatitis, perinatal transmission 2018 [
12. Schillie S, Vellozzi C, Reingold A, Harris A, Haber P, Ward JW, et al. Prevention of hepatitis B virus infection in the United States: recommendations of the Advisory Committee on Immunization Practices. MMWR Recommendations and Reports. 2018;67(1):1.
13. Shimakawa Y, Seck A, Nayagam S, Toure-Kane C, Lemoine M. Screening strategies to prevent mother-to-child transmission of hepatitis B in sub-Saharan Africa. The Lancet Gastroenterology & Hepatology. 2018;3(4):222-3.
14. Benhammou V, Tubiana R, Matheron S, Sellier P, Mandelbrot L, Chenadec JL, et al. HBV or HCV Coinfection in HIV-1-Infected Pregnant Women in France: Prevalence and Pregnancy Outcomes. Journal of acquired immune deficiency syndromes (1999). 2018;77(5):439-50.
15. Borgia G, Carleo MA, Gaeta GB, Gentile I. Hepatitis B in pregnancy. World Journal of Gastroenterology: WJG. 2012;18(34):4677.
16. Organization WH. Guidelines for the Prevention Care and Treatment of Persons with Chronic Hepatitis B Infection: Mar-15: World Health Organization; 2015.
17. Babatunde O, Smuts H, Eley B, Korsman S, De Lacy R, Hardie D. Fulminant hepatitis B virus (HBV) infection in an infant following mother-to-child transmission of an e-minus HBV mutant: Time to relook at HBV prophylaxis in South African infants. South African Medical Journal. 2018;108(5):389-92.
18. Israel GD. Determining sample size. 1992.
19. Organization WH. WHO guidelines on drawing blood: best practices in phlebotomy. 2010.
20. Zampino R, Boemio A, Sagnelli C, Alessio L, Adinolfi LE, Sagnelli E, et al. Hepatitis B virus burden in developing countries. World journal of gastroenterology. 2015;21(42):11941.
21. Pirillo MF, Bassani L, Germinario EA, Mancini MG, Vyankandondera J, Okong P, et al. Seroprevalence of hepatitis B and C viruses among HIV-infected pregnant women in Uganda and Rwanda. Journal of medical virology. 2007;79(12):1797-801.
22. Mutagoma M, Balisanga H, Malamba SS, Sebuhoro D, Remera E, Riedel DJ, et al. Hepatitis B virus and HIV co-infection among pregnant women in Rwanda. BMC infectious diseases. 2017;17(1):618.
23. Ngaira JAM, Kimotho J, Mirigi I, Osman S. Prevalence, awareness and risk factors associated with Hepatitis B infection among pregnant women attending the antenatal clinic at Mbagathi District Hospital in Nairobi, Kenya. The Pan African Medical Journal. 2016;24.
24. Chernet A, Yesuf A, Alagaw A. Seroprevalence of Hepatitis B virus surface antigen and factors associated among pregnant women in Dawuro zone, SNNPR, Southwest Ethiopia: a cross sectional study. BMC research notes. 2017;10(1):418.
25. Walker TY, Smith EA, Fenlon N, Lazaroff JE, Dusek C, Fineis P, et al. Characteristics of Pregnant Women With Hepatitis B Virus Infection in 5 US Public Health Jurisdictions, 2008-2012. Public Health Reports. 2016;131(5):685-94.

For peer review only - http://bmjopen.bmj.com/site/about/guidelines.xhtml

26. Visser M, van der Ploeg C, Smit C, Abbink F, Hukkelhoven C, Ommen P, et al. Evaluating progress towards triple elimination of mother-to-child transmission of HIV, syphilis and hepatitis B in the Netherlands, 2012-2015. 2018.
27. Abdhalah K Ziraba JB, Alice Namale, Caroline W Wainaina and Harriet Mayanja-Kizza. Sero-prevalence and risk factors for hepatitis B virus infection among health care workers in a tertiary hospital in Uganda. BMC Infectious Diseases. 2010;10(191).
28. Noubiap JJN, Nansseu JRN, Ndoula ST, Bigna JJR, Jingi AM, Fokom-Domgue J. Prevalence, infectivity and correlates of hepatitis B virus infection among pregnant women in a rural district of the Far North Region of Cameroon. BMC Public Health. 2015;15(1):454.
29. Souza MT, de Pinho TLR, Santos MDC, dos Santos A, Monteiro VL, Fonsêca LMB, et al. Prevalence of hepatitis B among pregnant women assisted at the public maternity hospitals of São Luís, Maranhão, Brazil. The Brazilian Journal of Infectious Diseases. 2012;16(6):517-20.
30. Utoo B. Hepatitis B surface antigenemia (HBsAg) among pregnant women in southern Nigeria. African health sciences. 2013;13(4):1139-43.
31. Manyahi J, Msigwa Y, Mhimbira F, Majigo M. High sero-prevalence of hepatitis B virus and human immunodeficiency virus infections among pregnant women attending antenatal clinic at Temeke municipal health facilities, Dar es salaam, Tanzania: a cross sectional study. BMC pregnancy and childbirth. 2017;17(1):109.
32. Umare A, Seyoum B, Gobena T, Mariyam TH. Hepatitis B virus infections and associated factors among pregnant women attending antenatal Care Clinic at Deder Hospital, eastern Ethiopia. PloS one. 2016;11(11):e0166936.
33. Kirbak ALS. Sero-prevalence for Hepatitis B virus among pregnant women attending antenatal clinic in Juba Teaching Hospital, Republic of South Sudan. The Pan African medical journal. 2017;26.
34. Buseri F, Seiyaboh E, Jeremiah Z. Surveying infections among pregnant women in the Niger Delta, Nigeria. Journal of Global infectious diseases. 2010;2(3):203.
35. Weinbaum CM, Williams I, Mast EE, Wang SA, Finelli L, Wasley A, et al. Recommendations for identification and public health management of persons with chronic hepatitis B virus infection. 2008.
36. WHO. Exposure to blood or other body fluids

2009 [
37. Organization WHO. Hepatitis B vaccines: WHO position paper, July 2017–Recommendations. Vaccine. 2019;37(2):223-5.

For peer review only - http://bmjopen.bmj.com/site/about/guidelines.xhtml

Reporting checklist for cross sectional study.

Based on the STROBE cross sectional guidelines.

Instructions to authors

Complete this checklist by entering the page numbers from your manuscript where readers will find each of the items listed below.

Your article may not currently address all the items on the checklist. Please modify your text to include the missing information. If you are certain that an item does not apply, please write "n/a" and provide a short explanation.

Upload your completed checklist as an extra file when you submit to a journal.

In your methods section, say that you used the STROBE cross sectionalreporting guidelines, and cite them as:

von Elm E, Altman DG, Egger M, Pocock SJ, Gotzsche PC, Vandenbroucke JP. The Strengthening the Reporting of Observational Studies in Epidemiology (STROBE) Statement: guidelines for reporting observational studies.

		Reporting Item	Page Number
**Title and abstract**			
Title	#1a	Indicate the study's design with a commonly used term in the title or the abstract	Title page
Abstract	#1b	Provide in the abstract an informative and balanced summary	Page i

For peer review only

		of what was done and what was found	

Introduction

Background / rationale	#2	Explain the scientific background and rationale for the investigation being reported	Page 1
Objectives	#3	State specific objectives, including any prespecified hypotheses	Page 2

Methods

Study design	#4	Present key elements of study design early in the paper	Page 3
Setting	#5	Describe the setting, locations, and relevant dates, including periods of recruitment, exposure, follow-up, and data collection	Page 3
Eligibility criteria	#6a	Give the eligibility criteria, and the sources and methods of selection of participants.	Pgs 3,4&5
	#7	Clearly define all outcomes, exposures, predictors, potential confounders, and effect modifiers. Give diagnostic criteria, if applicable	Page 4
Data sources / measurement	#8	For each variable of interest give sources of data and details of methods of assessment (measurement). Describe comparability of assessment methods if there is more than one group. Give information separately for exposed and unexposed groups if applicable.	Pgs 5 & 6
Bias	#9	Describe any efforts to address potential sources of bias	Page 7
Study size	#10	Explain how the study size was arrived at	Page 4

For peer review only

Quantitative variables	#11	Explain how quantitative variables were handled in the analyses. If applicable, describe which groupings were chosen, and why	Pages 6 & 7
Statistical methods	#12a	Describe all statistical methods, including those used to control for confounding	Pages 6 & 7
Statistical methods	#12b	Describe any methods used to examine subgroups and interactions	Pages 7 & 9
Statistical methods	#12c	Explain how missing data were addressed	Page 6
Statistical methods	#12d	If applicable, describe analytical methods taking account of sampling strategy	Pages 6, 7 & 9
Statistical methods	#12e	Describe any sensitivity analyses	Pages 6, 7 and 9
**Results**			
Participants	#13a	Report numbers of individuals at each stage of study—eg numbers potentially eligible, examined for eligibility, confirmed eligible, included in the study, completing follow-up, and analysed. Give information separately for for exposed and unexposed groups if applicable.	Page 5
Participants	#13b	Give reasons for non-participation at each stage	Page 5
Participants	#13c	Consider use of a flow diagram	n/a
Descriptive data	#14a	Give characteristics of study participants (eg demographic,	Page 8

For peer review only

		clinical, social) and information on exposures and potential confounders. Give information separately for exposed and unexposed groups if applicable.	
Descriptive data	#14b	Indicate number of participants with missing data for each variable of interest	n/a
Outcome data	#15	Report numbers of outcome events or summary measures. Give information separately for exposed and unexposed groups if applicable.	Page 8
Main results	#16a	Give unadjusted estimates and, if applicable, confounder-adjusted estimates and their precision (eg, 95% confidence interval). Make clear which confounders were adjusted for and why they were included	Pages 8 to 13
Main results	#16b	Report category boundaries when continuous variables were categorized	Pages 8 to 13
Main results	#16c	If relevant, consider translating estimates of relative risk into absolute risk for a meaningful time period	n/a
Other analyses	#17	Report other analyses done—e.g., analyses of subgroups and interactions, and sensitivity analyses	Pages 8 to 13
**Discussion**			
Key results	#18	Summarise key results with reference to study objectives	Pages 14 to 17
Limitations	#19	Discuss limitations of the study, taking into account sources of potential bias or imprecision. Discuss both direction and	Page ii and 17

For peer review only

		magnitude of any potential bias.	
Interpretation	#20	Give a cautious overall interpretation considering objectives, limitations, multiplicity of analyses, results from similar studies, and other relevant evidence.	Page 17
Generalisability	#21	Discuss the generalisability (external validity) of the study results	Page 17 and 18
**Other Information**			
Funding	#22	Give the source of funding and the role of the funders for the present study and, if applicable, for the original study on which the present article is based	Page ii

None The STROBE checklist is distributed under the terms of the Creative Commons Attribution License CC-BY. This checklist can be completed online using https://www.goodreports.org/, a tool made by the EQUATOR Network in collaboration with Penelope.ai

For peer review only

BMJ Open

Prevalence of Hepatitis B Virus infection and associated factors among pregnant women attending antenatal care clinic in Mulago Hospital, Uganda: A cross-sectional study.

Journal:	*BMJ Open*
Manuscript ID	bmjopen-2019-033043.R1
Article Type:	Original research
Date Submitted by the Author:	29-Mar-2020
Complete List of Authors:	Kayondo, Simon Peter; Makerere University College of Health Sciences, Obstetrics and Gynaecology; Association of Obstetricians and Gynaecologists of Uganda, Byamugisha, Josaphat; Makere University/Mulago National Referral Hospital, Obstetrics and Gynecology Ntuyo, Peter; Makere University/Mulago National Referral Hospital, Obstetrics and Gynecology
**Primary Subject Heading**:	Obstetrics and gynaecology
Secondary Subject Heading:	Obstetrics and gynaecology, Infectious diseases, Haematology (incl blood transfusion)
Keywords:	Hepatitis B, prevalence, pregnant women, Mulago hospital, Uganda

SCHOLARONE™
Manuscripts

I, the Submitting Author has the right to grant and does grant on behalf of all authors of the Work (as defined in the below author licence), an exclusive licence and/or a non-exclusive licence for contributions from authors who are: i) UK Crown employees; ii) where BMJ has agreed a CC-BY licence shall apply, and/or iii) in accordance with the terms applicable for US Federal Government officers or employees acting as part of their official duties; on a worldwide, perpetual, irrevocable, royalty-free basis to BMJ Publishing Group Ltd ("BMJ") its licensees and where the relevant Journal is co-owned by BMJ to the co-owners of the Journal, to publish the Work in this journal and any other BMJ products and to exploit all rights, as set out in our licence.

The Submitting Author accepts and understands that any supply made under these terms is made by BMJ to the Submitting Author unless you are acting as an employee on behalf of your employer or a postgraduate student of an affiliated institution which is paying any applicable article publishing charge ("APC") for Open Access articles. Where the Submitting Author wishes to make the Work available on an Open Access basis (and intends to pay the relevant APC), the terms of reuse of such Open Access shall be governed by a Creative Commons licence – details of these licences and which Creative Commons licence will apply to this Work are set out in our licence referred to above.

Other than as permitted in any relevant BMJ Author's Self Archiving Policies, I confirm this Work has not been accepted for publication elsewhere, is not being considered for publication elsewhere and does not duplicate material already published. I confirm all authors consent to publication of this Work and authorise the granting of this licence.

For peer review only

Prevalence of Hepatitis B Virus infection and associated factors among pregnant women attending antenatal care clinic in Mulago Hospital, Uganda: A cross-sectional study.

S.P. Kayondo[1], J. Byamugisha[2], P. Ntuyo[3].

Authors:

1. **Dr. Kayondo Simon Peter MBChB, M. Med Obs/Gyn (MUK)**
   Project Coordinator,
   Association of Obstetricians & Gynaecologists of Uganda (AOGU)
   P.O. Box 11966,
   Kampala- Uganda.
   Email: **kayondosimondr@gmail.com**
   Mobile: +256782736078

2. **Assoc. Prof. Josaphat Byamugisha, MBChB, M. Med Obs/Gyn, Ph.D. (MUK)**
   Associate Professor,
   Department of Obstetrics and Gynaecology,
   Makerere University College of Health Sciences,
   P.O. Box 7072**,**
   Kampala- Uganda**.**
   Email: **byamugisha2001@yahoo.com**
   Mobile: +256772580330

3. **Dr. Peter Ntuyo, MBChB, M. Med Obs/Gyn (MUK)**
   Obstetrician and Gynecologist,
   Directorate of Obstetrics and Gynecology,
   Mulago Hospital,
   P.O. Box 7051**,**
   Kampala- Uganda**.**
   Email: **peterntuyo@yahoo.com**
   Mobile: +256772481636.

Correspondence author:

Dr. Kayondo Simon Peter MBChB, M. Med Obs/Gyn (MUK)
Project Coordinator,
Association of Obstetricians & Gynaecologists of Uganda (AOGU)
P.O. Box 11966**,**
Kampala- Uganda**.**
Email: **kayondosimondr@gmail.com**
Mobile: +256782736078

For peer review only

ABSTRACT

Aim To determine the prevalence and factors associated with Hepatitis B Virus infection, among pregnant women attending antenatal care clinic in Mulago Hospital.

Design Cross-sectional observational study.

Setting Mulago National Referral Hospital, Uganda, Antenatal Care Clinic.

Participants We randomly selected 340 pregnant women attending their first antenatal care visit at Mulago Hospital Antenatal care clinic, into the study**.**

Primary Outcome Hepatitis B surface antigen (HBsAg) positivity.

Results We recruited 340 participants, with a mean age of 27±5.7 years, and a median gravidity of 3. The prevalence of Hepatitis B Virus infection among pregnant women attending the antenatal care clinic in Mulago Hospital, in our study, was 2.9% (N=10). (95% CI, 1.58-5.40). We found these factors associated with Hepatitis B virus infection, marital status, (aOR=11.37, p=0.002), history of having had a Hepatitis B positive patient at home, (aOR=49.52, p<0.001), and the history of having had a blood or body fluid splash to mucous membranes from a Hepatitis B positive patient, (aOR=61.69, p=0.015). Other factors such as age, socioeconomic status, number of sexual partners, HIV serostatus, piercing of ears, and history of blood transfusion, were not significantly associated with Hepatitis B virus infection in this study.

Conclusion The prevalence of Hepatitis B virus infection among pregnant women attending antenatal care clinic in Mulago Hospital, was of intermediate endemicity. We found marital status, history of having a Hepatitis B positive patient at home and history of a blood or body fluid splash to mucous membranes from a Hepatitis B positive patient, independently associated with Hepatitis B virus infection. Factors such as age, HIV status, history of blood transfusion, piercing of ears, and social status were of limited value in this study.

keywords: Hepatitis B, prevalence, pregnant women, Mulago Hospital, Uganda.

For peer review only - http://bmjopen.bmj.com/site/about/guidelines.xhtml

Strengths and limitations of this study.

- Qualified personnel followed standard laboratory procedures, at an ISO 9001 certified lab using a highly sensitive, and specific HBsAg test kit, FaStep.
- We ensured blinding of laboratory technologists through use of coded specimens.
- We did recruitment of the study participants using systematic random sampling procedure.
- We performed our study in a national referral and teaching hospital, meaning it may not accurately reflect the situation in other settings.
- Because of limited counts in our study, the confidence intervals in the statistical analysis for associated factors were wide.

Funding statement DELTAS Africa Initiative grant #DEL-15-011 to THRiVE-2 supported this work. The DELTAS Africa Initiative is an independent funding scheme of the African Academy of Sciences (AAS)'s Alliance for Accelerating Excellence in Science in Africa (AESA) and supported by the New Partnership for Africa's Development Planning and Coordinating Agency (NEPAD Agency) with funding from the Wellcome Trust grant #107742/Z/15/Z and the UK government. The views expressed in this publication are those of the authors and not necessarily those of AAS, NEPAD Agency, Wellcome Trust, or the UK government.

Conflict of interest: The authors declare no conflict of interest.

For peer review only

For peer review only - http://bmjopen.bmj.com/site/about/guidelines.xhtml

Introduction.

Hepatitis B is a viral infection of the liver that can cause acute and chronic disease. Worldwide, about two billion people are exposed to Hepatitis B virus, and about 257 million people are living with Hepatitis B virus infection (defined as HBsAg positive) (1). Hepatitis B virus infection resulted in 887,000 deaths in 2015, mostly from liver complications; liver cirrhosis and Hepatocellular Carcinoma. Hepatitis B prevalence is highest in the WHO Western Pacific Region, and the WHO African region, standing at 6.2% and 6.1% of the adult population, respectively. HBV infection and its sequelae are very common in Sub-Saharan Africa. 5-20%, is the overall HBsAg carrier rate in the general population, and is among the highest in the World. Perinatal transmission is estimated at 1-5% (2). 72% is the lifetime exposure in Uganda, with the national prevalence averaging about 10%. There's regional variation, however, the highest prevalence being in the northern part of the country, ranging from 19% in the North West to 25% in the North East (3, 4). In pregnant women, the prevalence statistics worldwide are missing. However, in Northern Uganda, prevalence was found to be about 11.8% (5). Namirembe et al. estimated prevalence at Mulago Hospital, at 0.9%, in 2016 (6), although this study had several limitations in participant recruitment, and estimation of mother to child transmission risk.

In highly endemic areas, transmission from mother to child at birth plays a key role in Hepatitis B virus infection. However, horizontal transmission, through exposure to infected blood mostly from an infected child to an uninfected child within the first five years of life, remains an issue (1, 7). Infants infected from their mothers, or before the age of five, often develop chronic infection (1, 8, 9). The transmission of Hepatitis B can also be through percutaneous or mucosal exposure to infected blood and various body fluids, such as saliva, menstrual, vaginal and seminal fluids(1, 10). Spread can also happen in men who have sex with men, through reuse of injections and in injectable drug users. The infection is also transmitted through unsafe medical, dental, and surgical procedures, and through practices such as tattooing, or use of sharp instruments contaminated with blood (1).

The risk of developing chronic Hepatitis B for babies born to a mother with Hepatitis B is greater than 90%, if we do not properly treat them at birth. It is therefore imperative for pregnant women to know their Hepatitis B status. We should test them early, in the first trimester (1). If tested positive, the baby, at birth, should receive both the first dose of the Hepatitis B vaccine, and the

For peer review only - http://bmjopen.bmj.com/site/about/guidelines.xhtml

Hepatitis B Immunoglobulin (a CDC recommendation), although it may not be available in all countries (1, 11, 12). If we give the two within the first 12 hours of life, the new-born has over a 90% chance of being protected against a lifelong Hepatitis B infection (11-13). Infant vaccination for Hepatitis B was introduced in Uganda in 2002 (4), as part of the Uganda National Expanded Programme for Immunisation (UNEPI) schedule, but has done little to eradicate the infections from the general population. All adults currently above 18 years were not beneficiaries of this, and remain a high-risk group.

Acute or chronic infection in pregnancy, in most cases, is similar to that in the general population, it neither increases mortality, nor yields teratogenic effects (14, 15). But with acute infection, there is a higher incidence of low birth weight and prematurity, than in the general population (15). In pregnancy, because of the shift in the Th1 to Th2 response, there is a depressed immune response against HBV. WHO defines Hepatitis B Virus infection in new-borns as HBsAg positivity 6 months after birth (1, 16). The risk of mother to child transmission is very high, in the absence of prophylaxis, varying with the HBeAg/anti-HBe status of the mother, 70-90% for HBeAg positive, 25% for HBeAg negative/HBeAb-negative mothers and 12% for HBeAg-negative/anti HBe positive mothers (13, 15, 17).

Study objectives.

General objective.

To determine the prevalence and factors associated with Hepatitis B Virus infection among pregnant women attending antenatal care clinic in Mulago Hospital.

Specific objective.

1. To determine the prevalence of HBV infection among pregnant women, attending ANC clinic in Mulago Hospital.
2. To establish the factors associated with HBV infection, among pregnant women attending ANC clinic, in Mulago Hospital.

Methods:

Study design and setting.

This was a cross-sectional study, conducted at Mulago Hospital antenatal care clinic. Mulago Hospital is in Kampala City, the capital of Uganda. It is a public, general and teaching hospital, with a bed capacity of over 1500 beds, founded in 1917. It is located on Mulago hill, approximately

For peer review only - http://bmjopen.bmj.com/site/about/guidelines.xhtml

5km Northeast of Kampala's central business district. The hospital's ANC unit attends to approximately 2500 patients per month, making a wide sampling population for the researcher. We purposively selected Mulago Hospital because, it is a National referral hospital for Uganda, that serves people from all ethnic groups, races, and regions of Uganda, and refugees, such as those from Rwanda, South Sudan and Congo. Uganda has a population of over 34 million people. The researcher was a Master student at this hospital, making the performance of the study convenient for him.

Kawempe General Hospital, in Kawempe Division, temporarily houses the Mulago Hospital ANC Clinic, as the main Mulago Hospital is undergoing major renovation. The clinic continues to offer preventive and curative services, such as health education, immunization, prenatal maternal and fetal monitoring, among others. The clinic serves between 200 and 250 ANC mothers per day, of whom about 100 are attending their first ANC visit. It runs on three days a week, Tuesday, Wednesday and Thursday. It serves first visit attendees on each of these days. We enrolled only mothers attending their first visit into the study, as the flow of first visit attendees in the ANC clinic was convenient for recruitment, unlike their repeat visit counterparts. We enrolled the par right after their health education session, as they awaited examination.

Study population.

The study population was pregnant women attending antenatal care clinic, between December 2018 and February 2019, in Mulago Hospital, Kawempe site.

Eligibility criteria.

Inclusion criteria:

All pregnant women who were attending their first ANC visit at Mulago ANC clinic and were willing to participate in the study. We enrolled all women above the age of 18 years, and those below this age, who we considered as emancipated minors.

Exclusion criteria:

Pregnant women who could not understand either English or Luganda. This is because these were the two languages used for the consenting process and administration of the questionnaires.

Research variables:

The dependent variable was HBV infection among pregnant women (HBsAg positivity).

For peer review only - http://bmjopen.bmj.com/site/about/guidelines.xhtml

The independent variables were the factors associated with the positive HBV status. These include the sociodemographic factors such as age, marital status, level of education, socio-economic status and sexual behavior. Others are Obstetric factors, such as gravidity and parity. Other factors are; history of blood transfusion, ear piercings, tattoos, injection drug use, history of caring for an HBV positive patient, immunization status, among others.

Sample size estimation.

We estimated the sample size for prevalence was using the Kish and Leslie formula, 1965 (18).

Using a prevalence from Northern Uganda of 11.8% (5)

The sample size, for prevalence, would be 160 women.

We based the sample size estimation for factors associated with Hepatitis B Virus infection in pregnancy, on the hypothesis that the prevalence of HBV would be higher among women below 20 years than among those who are above 20 years (Comparing HBV infection among younger women, versus older women), as found by Bayo et al. (5). We used the formula for comparing two proportions by Fleiss (REF: Fleiss, Statistical Methods for Rates and Proportions, formulas pages 3.18 & 3.19) and as applied in the OpenEpi Calculator accessed at:

http://www.openepi.com/SampleSize/SSCC.htm

Assuming power of 80%, type 1 error of 5% and the frequency of HBV of 20% among women 20 years and below and frequency of 8.7% among those over 20 years, based on a cross-sectional study by Bayo P et al., in Lacor and Gulu Hospitals, Northern Uganda (5)

Total sample size for associated factors was 340.

This sample size, 340, was larger than the initial 160 for prevalence estimate, and therefore, we used **340 pregnant women**.

Sampling procedure.

We used systematic random probability sampling method. We got participants during the day of data collection, after getting the register book for the antenatal care clinic attendance, and the booking cards for the first visit attendees. We only enrolled pregnant women attending their first antenatal visit at Mulago antenatal care clinic. We collected the booking cards for the first visit attendees as they came in to the clinic, on the day of data collection, and we requested every 10th card owner to take part in the study. With an average daily attendance of about 100 first ANC visit pregnant women, and the ANC clinic running three days a week, 12 days in 4 weeks, to collect

For peer review only - http://bmjopen.bmj.com/site/about/guidelines.xhtml

data over three months, and complete the sample size of 340 subjects, we enrolled every 10th woman into the study. (100/10= 10 participants/day, 10X12X3=360 participants in three months). If the 10th woman declined participation in the study, or was ineligible, we then considered the 11th.

Study Procedure.

We collected data between December 2018 and February 2019.

Pregnant women, who had come to the antenatal clinic for their first visit, made the sampling frame. This was because of the ease of recruitment after counselling. We used systematic random sampling technique to arrive at the sampling units. We collected the booking cards for the first ANC visit attendees as they came in to the clinic on the day of data collection, and we asked every 10th card owner to take part in the study. The antenatal clinic midwives, who were the research assistants, approached the participants, and explained the study purpose to them. They then screened them for eligibility and asked them if they were willing to participate in the study. They then got informed consent. We used interviewer-administered semi-structured questionnaires to get socio-demographic information such as; age, gestation age, gravidity, parity, education and occupation, and other factors that could influence infection with HBV, such as injection drug use, tattoos, family history of HBV infection, sexual history, among others. Obstetric factors like gravidity and parity, previous history of taking care of an HBV positive patient, immunization against HBV, were all assessed.

The laboratory technician aseptically collected four millilitres (4mls) of whole blood from the antecubital fossa, into BD Clot Activator Tube vacutainers (labelled with the participant's study number), using WHO (2010) guidelines on blood collection (19). He then took the blood to the Kawempe Hospital Laboratory, centrifuged, and transferred an aliquot of serum, about 2mls, into cryovials. He did immunochromatographic assay using HBsAg Rapid Test Strip, FaStep, to do HBsAg test on the samples following the manufacturer's instructions (supplementary file). This rapid test kit has a relative sensitivity of >99.9%, a relative specificity of 99.9%, and an accuracy of >99.9%. He carried out the HBsAg rapid tests, using the following procedure: He labelled a rapid test strip with the patient identifiers, and placed on a flat surface. He pipetted 3 drops of serum from the cryovial and applied to the sample pad of the test strip. He read the result after 15 minutes. A positive result was one where two-coloured bands appeared on the membrane, one in

For peer review only - http://bmjopen.bmj.com/site/about/guidelines.xhtml

the control region, and another in the test region. A negative result was one, where only one band appeared, in the control region, and none in the test region. An invalid result was one where a band failed to appear in the control region.

We produced results and gave a copy to the patient after being counselled by the principal investigator, and the nurse counsellor. We sent all HBsAg positive samples to MBN Clinical Laboratories, Kampala, an ISO accredited laboratory, for HBsAg retesting for confirmation, HBeAg testing, and liver function tests. We took 5% of all HBsAg negative samples to MBN, for external quality assurance. We packed and transported all samples at 2-8 degrees Celsius, on the same day of collection. We referred HBV positive mothers to the Hepatitis B Clinic in Mulago Hospital (Kiruddu) for further care, where the physicians did their viral load assays, and further assessed them for treatment. We made a plan to vaccinate their babies within 24 hours after birth with the Hepatitis B Vaccine and coordinated it through the Mulago Hospital ANC clinic. (The study endeavoured to meet the cost of the HepB-BD vaccine, for the positive mothers).

Data Management and Analysis.

The principal investigator doubled raw data entry into Epidata 3.1 and imported into STATA 14.1.

We determined descriptive statistics such as mean, standard deviation and percentages. We presented results numerically as frequencies, percentages in charts and tables. We determined HBV prevalence that is the number of HBsAg positive pregnant women, divided by the number of all women recruited into the study. To assess factors associated with Hepatitis B virus infection in pregnancy, we performed bivariate analysis, to compute crude odds ratios at the 95% level of significance. At bivariate analysis, the t-test was used to analyse continuous variables such as age, while the Fischer's exact test was used to analyse categorical variables.

To assess the independent association of these risk factors, we performed multivariate analysis, where we considered all independent variables with a p-value of less than 0.2 at bivariate analysis, and those with biological significance, for multivariate logistic regression. At multivariate analysis, stepwise regression method was used to arrive at the model of best fit. We considered all

For peer review only - http://bmjopen.bmj.com/site/about/guidelines.xhtml

associations with p-value as adjusted Odds Ratio of 95% Confidence Interval, statistically significant. We presented the results of the analysis on tables.

Ethical considerations.

We got permission to carry out the study from the Department of Obstetrics and Gynaecology, Makerere University, and Makerere University School of Medicine Research and Ethics Committee, **(#REC REF 2018-119),** Mulago Hospital Research and Ethics Committee, **(#MHREC1519**), and the Uganda National Council for Science and Technology, **(#HS257ES),** before any collection of samples and data.

We explained information regarding the study to the subjects and got written consent for participation in the study in English/Luganda. We interviewed participants and collected samples, following local guidelines in privacy. Only study numbers, with unique participant identifiers, were used to label the questionnaires and the samples, and we used no names on questionnaires or samples. We kept filled questionnaires separate from signed consent forms and locked them away securely with only access to study team. Mothers had an informed consent taken, after we explained to them the study aim, procedures risks and benefit in a language they understood well. They could also drop out at any point of this study, with an assurance that if they did so, we would not deny them any other ANC services. We allowed mothers below the age of 18 to consent, as these are emancipated minors.

We only enrolled fully immunized Hepatitis B negative health workers, as research assistants. And they wore protective gear, such as gloves, masks, and eye glasses during laboratory procedures. These measures were to protect both mothers and health workers from acquiring Hepatitis B virus infection from each other during the study.

Patient and Public involvement While there were patients involved in this study as study participants, we did not involve them, at the stage of the study design. But the intended outcome in favour of the patient was that we made available Hepatitis B birth dose vaccination for all exposed babies, which is in the patients' interest. We circulated a summary of the findings using SMS messaging to the phone contacts of all study participants.

For peer review only - http://bmjopen.bmj.com/site/about/guidelines.xhtml

Results.

Description of the study population.

We recruited 340 participants into the study, with a mean age of 27±5.7 years (table 1). More than half, (56.8%), were from Kampala, 40% from Wakiso, and 3.2% from elsewhere**.** Most of the participants, (76.7%) had a secondary education and above, and 52.4% were employed. A little over a half of the participants, (51.7%), were business people, 41.5% in professional jobs, 5.1% casual laborers, while 3% were health workers. More than half of them (65.3%), were of low socioeconomic status, earning less than Uganda shillings 500,000 per month. Majority of the participants (70.3%), had had their sexual debut during their teenage years.

Table 1: Table showing demographic/gynecological/obstetric/sexual characteristics of the study participants.

**Demographic Characteristics.**	**Freq [N=340, (%)]**
**Age (years); mean (std. dev)**	27 (5.7)
**Age (years) categorized.**	
≤20	28 (8.2)
>20	312 (91.8)
**Employment status.**	
Yes	178 (52.4)
No	162 (47.6)
**Marital status.**	
Staying with a partner.	304 (89.4)
No stable partner.	36 (10.6)
**Gyn/Obs/Sexual characteristics.**	
**Gravidity; median (IQR)**	3 (2 - 3)
**Gravidity categorized.**	
1	67 (19.7)
2-4	211 (62.1)
>4	62 (18.2)
**Weeks of amenorrhea (WOA) categorized.**	
>20	211 (62.1)
≤20	54 (15.9)
Unknown	75 (22.1)
**Mother Hepatitis B positive?**	
Yes	4 (1.2)
No	126 (37.1)
I don't know.	210 (61.7)
**Number of sex partners; median (IQR)**	2 (1-3)
**Number of sex partners categorized.**	
1	117 (34.6)
≥2	221 (65.4)

Over half of the participants were employed, the majority (89.4%) were staying with a partner, and most of them had more than one lifetime sexual partners (table 1).

For peer review only - http://bmjopen.bmj.com/site/about/guidelines.xhtml

Factors associated with Hepatitis B virus infection.

Table 2: Table showing immunosuppression characteristics, vaccination history, familial exposure, occupational exposure risk of study participants, and exposure to Hepatitis B through sharp instruments, medical and surgical procedures.

	Freq [N=340, (%)]
**Immunosuppression characteristics.**	
**HIV sero-status**	
Positive	10 (2.9)
**History of Diabetes mellitus.**	
Yes	3 (0.9)
No	47 (13.8)
Unknown	290 (85.3)
**Vaccination history.**	
**Hepatitis B vaccination in the first year of life.**	
Yes	9 (2.6)
No	240 (70.6)
Don't know.	91 (26.8)
**Hepatitis B vaccination after the age of 10 years.**	
Yes	12 (3.5)
No	299 (88.0)
Don't know.	29 (8.5)
**Exposure through sharp instruments/medical/surgical procedures.**	
**Ever had a blood transfusion.**	
Yes	18 (5.3)
**Ever pierced your ears.**	
Yes	275 (80.9)
**Familial exposure**	
**Have a Hepatitis B positive patient at home?**	
Yes	7 (2.1)
No	291 (85.6)
Don't know.	42 (12.3)
**Occupational exposure.**	
**Ever had needle stick injury?**	
Yes	4 (1.2)
**Blood or body fluid splash from Hepatitis B positive patient.**	
Yes	2 (0.6)

Only 2.9% of study participants were HIV positive (table 2). Vaccination against Hepatitis B was low, with 2.6%, and 3.5% as the childhood and adult vaccination rates, respectively (table 2). About 2.1% of the participants reported having a Hepatitis B positive patient at home (table 2).

For peer review only - http://bmjopen.bmj.com/site/about/guidelines.xhtml

Prevalence
10 out of 340 participants were HBsAg positive, comprising 2.9%. (95% CI, 1.58-5.40)

All the HBsAg positive participants were HBeAg negative and had normal liver function tests.

Associations between the independent variables and Hepatitis B status.

Table 3: Table showing association between demographic, gynecological, obstetric characteristics and Hepatitis B status.

**Demographic characteristics.**	**Hepatitis B status.**		**Unadj Odds (95% CI)**	**P-value.**
	**Pos [n=10, (%)]**	**Neg [n=330, (%)]**		
**Age (years); mean (std. dev)**	27.4 (6.2)	26.9 (5.7)		**0.775[a]**
**Age (years) categorised.**				**0.582**
<20	1 (10.0)	27 (8.2)	1.00	
≥20	9 (90.0)	303 (91.8)	1.25 (0.15 - 10.21)	
**Employment status.**				**0.753**
Yes	6(60)	172(52.2)	1.00	
No	4(40)	158(47.8)	0.73(0.20-2.62)	
**Marital status.**				**0.004**
Staying with a partner.	6 (60.0)	298 (90.3)	1.00	**0.014**
No stable partner.	4 (40.0)	32 (9.7)	6.21 (1.66 - 23.16)	
**Gynecological/Obstetric factors.**				
**WOA categorized.**				**<0.001**
>20	1 (10.0)	210 (63.6)	1.00	
≤20	6 (60.0)	48 (14.6)	26.25 (3.09 - 223.13)	
Unknown	3 (30.0)	72 (21.8)	8.75 (0.90 - 85.46)	
**Mother Hepatitis B positive?**				**0.036**
Yes	1 (10.0)	3 (0.9)	1.00	
No	1 (10.0)	125 (37.9)	0.02 (0.00 - 0.48)	
I don't know.	8 (80.0)	202 (61.2)	0.12 (0.01 - 1.27)	

P- Fishers exact p-values, P[a]- t-test p-values.

At bivariate analysis, we found the participant's marital status, weeks of amenorrhea, mother's Hepatitis B status, significantly associated with Hepatitis B status (table 3), and we took these to multivariate analysis, along with age, for biological significance.

For peer review only - http://bmjopen.bmj.com/site/about/guidelines.xhtml

Table 4: Association of immunosuppression characteristics/occupational exposure risk/surgical procedures/sharp instruments and familial exposure with Hepatitis B status.

Immunosuppression characteristics.	Hepatitis B Status.		Unadjusted Odds (95% CI)	P-value.
	Pos [n=10, (%)]	Neg [n=330, (%)]		
**HIV sero-status**				**0.261**
Positive	1 (10.0)	9 (2.7)	3.96 (0.45—34.69)	
Negative	9 (90.0)	321 (97.3)	1.00	
**Occupational exposure risk.**				
**Blood or body fluid splash from a Hepatitis B positive patient.**				**0.058**
Yes	1(10)	1(0.3)	36.56 (2.11—631.85)	
No	9(90)	329(99.7)	1.00	
**Exposure through surgical procedures/sharp instruments.**				
**Ever had a blood transfusion.**				**0.424**
Yes	1 (10.0)	17 (5.2)	2.05 (0.24—17.09)	
No	9 (90.0)	313 (94.8)	1.00	
**Ever pierced your ears.**				**0.411**
Yes	7 (70.0)	268 (81.2)	1.00	
No	3 (30.0)	62 (18.8)	1.85 (0.47—7.37)	
**Familial exposure**				
**Have a Hepatitis B positive patient at home?**				**0.011**
Yes	2 (20.0)	5 (1.5)	1.00	
No	6 (60.0)	285 (86.4)	19.00 (3.05 - 118.24)	
Don't know.	2 (20.0)	40 (12.1)	2.38 (0.46 – 12.17)	

Having had a blood or body fluid splash to the mucous membranes from a Hepatitis B positive patient, and having a Hepatitis B positive patient at home were significantly associated with Hepatitis B status, at bivariate analysis (table 4). We took both to multivariate logistic regression analysis.

For peer review only - http://bmjopen.bmj.com/site/about/guidelines.xhtml

Table 5: Multivariable logistic regression model showing factors associated with Hepatitis B virus infection among study participants.

Characteristics	Adjusted Odds ratio (95% CI)	P-value
**Age(years) categorized**		
<20	1.00	
≥20	0.27 (0.01 - 5.31)	**0.387**
**Marital status**		
Staying with a partner	1.00	
No stable partner	11.37 (2.37 - 54.60)	**0.002**
**Have a Hepatitis B positive patient at home**		
No	1.00	
Yes	49.52 (6.24 - 392.85)	**<0.001**
Don't know.	4.25 (0.71 - 25.35)	**0.113**
**Blood or body fluid splash from Hepatitis B positive patient**		
No	1.00	
Yes	61.69 (2.24 - 1701.54)	**0.015**

We took variables from bivariate analysis with a p-value less than 0.2 to multivariable logistic regression analysis. We did Individual logistic regressions on each of these, and we adjusted variables with p-values less than 0.05 into a final logistic regression model. We arrived at the final model of best fit using the stepwise regression method.
We found marital status, having had a Hepatitis B positive patient at home, and history of a blood or body splash to mucous membranes from a Hepatitis B positive patient, significantly independently associated with Hepatitis B Virus infection, with p values < 0.05 (table 5). The association with age was not statistically significant (table 5).

Data availability statement: We have included all data relevant to this study in the article or uploaded them as supplementary information. Raw data is accessible on: https://drive.google.com/drive/folders/1wc0C3bP4Qge0OKtG4z-zXC6pbD1F3nsL?usp=sharing.

Discussion
We conducted this study to determine the prevalence and factors associated with Hepatitis B Virus infection, among pregnant women attending the antenatal care clinic in Mulago Hospital, a multicultural, multiracial national referral hospital.
Although Namirembe et al., in early 2016, estimated the prevalence of Hepatitis B virus among pregnant women at 0.9%, before Mulago Hospital was transferred to the new Kawempe site (6), their study was not without limitations, hence necessitating our study, to address these. Notably, their prevalence was much lower, compared to our 2.9%. Apart from having a different socio-

For peer review only - http://bmjopen.bmj.com/site/about/guidelines.xhtml

demographic profile, the 2016 study excluded pregnant women below 14 years, and above 43 years. It also excluded all participants who were bedridden, and all those with obstetric emergencies. This could have limited participants, and especially the likely positives, such as those who end up admitted. The other explanation for the difference may be because Kawempe, the site at which we performed our study, is a slum area, with many risky activities such as prostitution, increasing the risk profile of participants, as compared to the old Mulago site, where the 2016 study was done. Nonetheless, since majority (60%) of all positive pregnant women present in the first half of the pregnancy, as shown by our study (table 3), it is an opportunity for early testing and intervention to reduce MTCT.

The prevalence in our study, 2.9% (N=10) (95% CI, 1.58-5.40), lies in the Hepatitis B intermediate endemicity WHO category (2-8%) (20). It is comparable to the one found by Pirillo et al. in Kampala (Uganda), 4.9% and Kigali (Rwanda), 2.4%, in 2007 (21), and by Mutagoma et al. in Rwanda, 3.7% (22). Their study populations were different, however, as they did their studies among HIV positive pregnant women only, whereas ours was among both HIV positive and negative pregnant women. Other studies by Ngaira J et al. in Mbagathi district in Kenya, and by Chernet et al. in Ethiopia, found the prevalence of Hepatitis B among pregnant women attending Antenatal care to be 3.8%, and 3.5% respectively, which are still comparable to the one found in this study, and in a similar setting (23, 24). It is, however, higher than in low endemicity areas, such as the United States of America, at 0.38% found by Walker et al. (25). These low endemicity areas have vigorously implemented Hepatitis B prevention strategies, such as; universal testing of all pregnant women for Hepatitis B, universal birth dose vaccination and universal childhood immunization; unlike intermediate and high endemicity areas (26).
The 11.8% prevalence found by Bayo et al. in highly endemic Northern Uganda, among pregnant women in 2014(5), and the average National prevalence of about 10% in the general population, as estimated by Bwogi et al. from a National survey in 2009(4), are both higher than the prevalence in our study. The same holds for prevalence found in other select populations such as health workers in Mulago Hospital, found at 8.1% by Ziraba K. et al. (27). The explanation for the difference could be multi-pronged. The Central region is a low prevalence area, compared to other regions of Uganda, such as the North and East. There has also been an intensification of prevention programmes against Hepatitis B in the country, over the last few years, such as increased awareness, on the mode of spread and prevention. These all could have contributed to a reduction in prevalence of Hepatitis B in the general population, and hence among pregnant women.

In our study, we found marital status, history of having had a Hepatitis B positive patient at home, and history of having had a blood or body fluid splash from a Hepatitis B positive patient, as factors independently associated with Hepatitis B virus infection (table 5). Other factors that have been found to be significant in studies elsewhere, such as age (5, 25, 28, 29), level of education, (29, 30) socioeconomic status (31), number of sexual partners (24, 32), piercing of ears (32), history of abortions (23, 24), history of blood transfusion (28), and history of jaundice (33), among others, we did not find significantly associated with Hepatitis B virus infection in this study. HIV serostatus, a factor that several studies have found associated with HBV infection

For peer review only - http://bmjopen.bmj.com/site/about/guidelines.xhtml

(28, 31), did not have statistical significance in our study (table 4). This could be because the HIV prevalence in our study was generally low (table 2).

Women who did not have a stable partner, were eleven (11) times as likely to be Hepatitis B positive, as their counterparts who were staying with partners. (aOR=11.37, 95% CI, (2.37-54.60), p= 0.002, table 5). Kirbak et al. noted an almost similar finding in Juba, South Sudan, although the increased risk was specific to loss of a partner; (OR 4.4, CI 1.4 -13.9) (33). Chernet et al., and Umare et al. also found an association between multiple sexual partners and Hepatitis B positivity among pregnant women in Ethiopia, (aOR=6.923) and (aOR=16.8), respectively (24, 32). Whereas this is a little different from the lack of a stable partner, it outlines the risk associated with having no stable partner. On the contrary, Ngaira et al. in Kenya, did not find any significant association between marital status and Hepatitis B status, (23) and so did by Buseri et al., in Niger state, Nigeria (34). In our study, the women with no stable partner were widowed, separated, or unmarried. This increases the risk of having contracted Hepatitis B from the lost partner, or which increases risky behaviour, leading to higher chances of contracting the Hepatitis B virus.

Study participants who reported a history of having had a patient at home positive for Hepatitis B Virus, were almost fifty (50) times more likely to be positive for Hepatitis B, than those who did not (aOR=49.52, 95% CI, (6.24-392.85), p<0.001, table 5). CDC supports this finding, showing that 3-20% of people in contact with Chronic Hepatitis B patients have been found with evidence of Chronic Hepatitis B infection themselves, thus calling for screening and vaccination of all these contacts (35). Kirbak et al. found in Juba, South Sudan, also showed an increased risk of Hepatitis B infection among pregnant women who reported a history of loss of a partner (OR=4.4) (33). This important finding underscores the risk of horizontal transmission of Hepatitis B virus infection, either sexually between partners, or through contact with blood and body fluids, among people staying in the same home. This can be through sharing household items such as toothbrushes, razors, syringes and needles. The risk posed by having to take care of a Hepatitis B positive patient is even higher, with exposure to blood and body fluids infected with the virus. However, if the Hepatitis B positive patient at home was the mother, the risk of vertical transmission holds, with chances of the participants having contracted the infection from their mothers at birth. It is essential that those who live with, or take care of, a Hepatitis B positive patient be fully vaccinated against the same, to reduce the risk. And we should test all mothers during ANC and vertical transmission prevented through birth dose Hepatitis B vaccination of the exposed infant, within the first 24 hours.

Those who had ever had a blood or body fluid splash to mucous membranes from a Hepatitis B positive patient, were almost 62 times more likely to be Hepatitis B positive than those who had never had that experience (aOR=61.69, 95% CI, (2.24-1701.54), p=0.015, table 5). A study by Ziraba et al. among health workers in Mulago Hospital, which reported an 8.1% prevalence of Hepatitis B in this population, showed that exposure to potentially infectious body fluids was high in this population, and yet a small percentage of the health workers were vaccinated (27). This therefore is still a significant route of potential infection with the Hepatitis B virus, be it from an infected patient to a health worker, or from a Hepatitis B positive patient to their caretaker, especially if the health worker or the caretaker are not vaccinated against Hepatitis B.

For peer review only - http://bmjopen.bmj.com/site/about/guidelines.xhtml

WHO estimates the risk of infection with Hepatitis B after a single percutaneous exposure to be 6-60%, which is higher than Hepatitis C, 2%, and HIV, 0.1-0.3% (36) It is not a surprise then, that some Hepatitis B positive participants in our study were health workers, and some admitted to this risk, while others had nursed a Hepatitis B positive patient in their lifetime, with a similar risk. Therefore, the need to vaccinate especially health workers and people in contact with patients positive for Hepatitis B remains paramount (37).

The notable limitations of our study are the fact that we performed it in a national referral and teaching hospital, meaning it may not accurately reflect the situation in other settings; and that because of the limited counts in our study, the confidence intervals in the statistical analysis for associated factors were wide.

Conclusion

The prevalence of Hepatitis B virus infection among pregnant women attending antenatal care clinic in Mulago Hospital was of intermediate endemicity.

We found marital status, history of having had a Hepatitis B positive patient at home, and history of a blood or body fluid splash to mucous membranes from a Hepatitis B positive patient, independently associated with Hepatitis B Virus infection.

Factors such as age, HIV status, history of blood transfusion, piercing of ears, and social status were of limited value in this study.

Recommendations

Because of the intermediate endemicity prevalence, we should test all pregnant women for Hepatitis B virus infection during antenatal care. For all babies born to Hepatitis B positive mothers, birth dose Hepatitis B vaccine, with or without Hepatitis B Immunoglobulin for all, followed with two other doses within the first six months of life, should be given, to curb mother to child transmission.

Since most of the adult population did not get childhood vaccination, which was only introduced in Uganda in 2002, we recommend screening and adult vaccination with 3 doses of Hepatitis B vaccine for high-risk groups, such as health workers, people living or taking care of Hepatitis B positive patients, and women without stable partners, to control horizontal transmission.

We further recommend vaccination of pregnant women with the three doses of the Hepatitis B vaccine, within six months, seeing as it is not contraindicated in pregnancy, and yet protective.

Additionally, there should be clear referral pathways for Hepatitis B positive mothers to access specialist care for further serological, virologic and hepatological assessment and management, including long term monitoring.

Finally, we recommend a wider study, preferably multicentre, with a larger sample size, and a wider variety of tests, such as viral load, Anti-Hepatitis B Core Antibody, in this same study population. This will find out the exposure status to Hepatitis B virus, the risk of vertical transmission, and more generalizable associated factors.

For peer review only - http://bmjopen.bmj.com/site/about/guidelines.xhtml

Authors' contributions.

KSP developed the study's concept, designed the study methods, and also took part in data collection, analysis and interpretation. PN revised the manuscript critically and made amendments for important intellectual content; JB contributed significantly to the intellectual content of the manuscript and also gave the final approval of the version submitted. All the authors have agreed to be accountable for all aspects of the work in this study.

Authors' information.

KSP is an Obstetrician and Gynaecologist Department of Obstetrics and Gynaecology, Makerere University College of Health Sciences. PN is an Obstetrician and Gynaecologist, Directorate of Obstetrics and Gynaecology, Mulago Hospital. JB is an Associate Professor, Department of Obstetrics and Gynaecology, Makerere University College of Health Sciences.

Acknowledgements

We thank all senior members of the Department of Obstetrics and Gynaecology, Makerere University and members of the Directorate of Obstetrics and Gynaecology, Mulago Hospital, for the support and direction during the study. Hastily, we thank Assoc. Prof. Annettee Nakimuli, for her tremendous input to this study and providing a conducive environment for us to carry it out. Great thanks to Mr. Godwin Anguzu, who offered technical input during data analysis. Finally, great thanks to the whole dedicated data collection team in the Mulago Hospital antenatal care clinic, Kawempe site.

REFERENCES

1. Organisation WHO. Hepatitis B Fact Sheet World Health Organisation; 2018 [

2. Kiire C, Group ARS. Hepatitis B infection in sub-Saharan Africa. Vaccine. 1990;8:S107-S12.
3. Ochola E, Ocama P, Orach CG, Nankinga ZK, Kalyango JN, McFarland W, et al. High burden of hepatitis B infection in Northern Uganda: results of a population-based survey. BMC Public Health. 2013;13(1):727.
4. Bwogi J, Braka F, Makumbi I, Mishra V, Bakamutumaho B, Nanyunja M, et al. Hepatitis B infection is highly endemic in Uganda: findings from a national serosurvey. African health sciences. 2009;9(2).
5. Bayo P, Ochola E, Oleo C, Mwaka AD. High prevalence of hepatitis B virus infection among pregnant women attending antenatal care: a cross-sectional study in two hospitals in northern Uganda. BMJ Open. 2014;4(11):e005889.
6. Allen N, Bashir M, Mugisha TI. Prevalence and Associated Factors of Hepatitis B Virus Infection among Pregnant Women Attending Antenatal Care Clinic at Mulago National Referral Hospital, Uganda. International Blood Research & Reviews. 2017:1-10.
7. Paul T, Marie TP, Bechem E. Knowledge, attitude and practice of staff of 4 hospitals in Yaoundé on the prevention of vertical transmission of hepatitis B. Pan African Medical Journal. 2017;28(1).
8. Ifeorah I, Bakarey A, Adewumi M, Faleye T, Akere A, Omoruyi C, et al. Patterns of serologic markers of hepatitis B virus infection and the risk of transmission among pregnant women in Southwestern Nigeria. Journal of Immunoassay and Immunochemistry. 2017;38(6):639-51.
9. Shi Y-H, Shi C-H. Molecular characteristics and stages of chronic hepatitis B virus infection. World journal of gastroenterology: WJG. 2009;15(25):3099.

For peer review only - http://bmjopen.bmj.com/site/about/guidelines.xhtml

10. Bodo B, Malande OO. Delayed introduction of the birth dose of Hepatitis B vaccine in EPI programs in East Africa: a missed opportunity for combating vertical transmission of Hepatitis B. The Pan African Medical Journal. 2017;27(Suppl 3).
11. Prevention CfDCa. Viral hepatitis, perinatal transmission 2018 [
12. Schillie S, Vellozzi C, Reingold A, Harris A, Haber P, Ward JW, et al. Prevention of hepatitis B virus infection in the United States: recommendations of the Advisory Committee on Immunization Practices. MMWR Recommendations and Reports. 2018;67(1):1.
13. Shimakawa Y, Seck A, Nayagam S, Toure-Kane C, Lemoine M. Screening strategies to prevent mother-to-child transmission of hepatitis B in sub-Saharan Africa. The Lancet Gastroenterology & Hepatology. 2018;3(4):222-3.
14. Benhammou V, Tubiana R, Matheron S, Sellier P, Mandelbrot L, Chenadec JL, et al. HBV or HCV Coinfection in HIV-1-Infected Pregnant Women in France: Prevalence and Pregnancy Outcomes. Journal of acquired immune deficiency syndromes (1999). 2018;77(5):439-50.
15. Borgia G, Carleo MA, Gaeta GB, Gentile I. Hepatitis B in pregnancy. World Journal of Gastroenterology: WJG. 2012;18(34):4677.
16. Organization WHO. Guidelines for the Prevention Care and Treatment of Persons with Chronic Hepatitis B Infection: Mar-15: World Health Organization; 2015.
17. Babatunde O, Smuts H, Eley B, Korsman S, De Lacy R, Hardie D. Fulminant hepatitis B virus (HBV) infection in an infant following mother-to-child transmission of an e-minus HBV mutant: Time to relook at HBV prophylaxis in South African infants. South African Medical Journal. 2018;108(5):389-92.
18. Israel GD. Determining sample size. 1992.
19. Organization WH. WHO guidelines on drawing blood: best practices in phlebotomy. 2010.
20. Zampino R, Boemio A, Sagnelli C, Alessio L, Adinolfi LE, Sagnelli E, et al. Hepatitis B virus burden in developing countries. World journal of gastroenterology. 2015;21(42):11941.
21. Pirillo MF, Bassani L, Germinario EA, Mancini MG, Vyankandondera J, Okong P, et al. Seroprevalence of hepatitis B and C viruses among HIV-infected pregnant women in Uganda and Rwanda. Journal of medical virology. 2007;79(12):1797-801.
22. Mutagoma M, Balisanga H, Malamba SS, Sebuhoro D, Remera E, Riedel DJ, et al. Hepatitis B virus and HIV co-infection among pregnant women in Rwanda. BMC infectious diseases. 2017;17(1):618.
23. Ngaira JAM, Kimotho J, Mirigi I, Osman S. Prevalence, awareness and risk factors associated with Hepatitis B infection among pregnant women attending the antenatal clinic at Mbagathi District Hospital in Nairobi, Kenya. The Pan African Medical Journal. 2016;24.
24. Chernet A, Yesuf A, Alagaw A. Seroprevalence of Hepatitis B virus surface antigen and factors associated among pregnant women in Dawuro zone, SNNPR, Southwest Ethiopia: a cross sectional study. BMC research notes. 2017;10(1):418.
25. Walker TY, Smith EA, Fenlon N, Lazaroff JE, Dusek C, Fineis P, et al. Characteristics of Pregnant Women With Hepatitis B Virus Infection in 5 US Public Health Jurisdictions, 2008-2012. Public Health Reports. 2016;131(5):685-94.
26. Visser M, van der Ploeg C, Smit C, Abbink F, Hukkelhoven C, Ommen P, et al. Evaluating progress towards triple elimination of mother-to-child transmission of HIV, syphilis and hepatitis B in the Netherlands, 2012-2015. 2018.
27. Abdhalah K Ziraba JB, Alice Namale, Caroline W Wainaina and Harriet Mayanja-Kizza. Sero-prevalence and risk factors for hepatitis B virus infection among health care workers in a tertiary hospital in Uganda. BMC Infectious Diseases. 2010;10(191).
28. Noubiap JJN, Nansseu JRN, Ndoula ST, Bigna JJR, Jingi AM, Fokom-Domgue J. Prevalence, infectivity and correlates of hepatitis B virus infection among pregnant women in a rural district of the Far North Region of Cameroon. BMC Public Health. 2015;15(1):454.

For peer review only - http://bmjopen.bmj.com/site/about/guidelines.xhtml

29. Souza MT, de Pinho TLR, Santos MDC, dos Santos A, Monteiro VL, Fonsêca LMB, et al. Prevalence of hepatitis B among pregnant women assisted at the public maternity hospitals of São Luís, Maranhão, Brazil. The Brazilian Journal of Infectious Diseases. 2012;16(6):517-20.
30. Utoo B. Hepatitis B surface antigenemia (HBsAg) among pregnant women in southern Nigeria. African health sciences. 2013;13(4):1139-43.
31. Manyahi J, Msigwa Y, Mhimbira F, Majigo M. High sero-prevalence of hepatitis B virus and human immunodeficiency virus infections among pregnant women attending antenatal clinic at Temeke municipal health facilities, Dar es salaam, Tanzania: a cross sectional study. BMC pregnancy and childbirth. 2017;17(1):109.
32. Umare A, Seyoum B, Gobena T, Mariyam TH. Hepatitis B virus infections and associated factors among pregnant women attending antenatal Care Clinic at Deder Hospital, eastern Ethiopia. PloS one. 2016;11(11):e0166936.
33. Kirbak ALS. Sero-prevalence for Hepatitis B virus among pregnant women attending antenatal clinic in Juba Teaching Hospital, Republic of South Sudan. The Pan African medical journal. 2017;26.
34. Buseri F, Seiyaboh E, Jeremiah Z. Surveying infections among pregnant women in the Niger Delta, Nigeria. Journal of Global infectious diseases. 2010;2(3):203.
35. Weinbaum CM, Williams I, Mast EE, Wang SA, Finelli L, Wasley A, et al. Recommendations for identification and public health management of persons with chronic hepatitis B virus infection. 2008.
36. WHO. Exposure to blood or other body fluids

2009 [
37. Organization WHO. Hepatitis B vaccines: WHO position paper, July 2017–Recommendations. Vaccine. 2019;37(2):223-5.

For peer review only - http://bmjopen.bmj.com/site/about/guidelines.xhtml

Reporting checklist for cross sectional study.

Based on the STROBE cross sectional guidelines.

Instructions to authors

Complete this checklist by entering the page numbers from your manuscript where readers will find each of the items listed below.

Your article may not currently address all the items on the checklist. Please modify your text to include the missing information. If you are certain that an item does not apply, please write "n/a" and provide a short explanation.

Upload your completed checklist as an extra file when you submit to a journal.

In your methods section, say that you used the STROBE cross sectionalreporting guidelines, and cite them as:

von Elm E, Altman DG, Egger M, Pocock SJ, Gotzsche PC, Vandenbroucke JP. The Strengthening the Reporting of Observational Studies in Epidemiology (STROBE) Statement: guidelines for reporting observational studies.

		Reporting Item	Page Number
**Title and abstract**			
Title	#1a	Indicate the study's design with a commonly used term in the title or the abstract	Title page
Abstract	#1b	Provide in the abstract an informative and balanced summary	Page i

For peer review only

		of what was done and what was found	
**Introduction**			
Background / rationale	#2	Explain the scientific background and rationale for the investigation being reported	Pages 1&2
Objectives	#3	State specific objectives, including any prespecified hypotheses	Page 2
**Methods**			
Study design	#4	Present key elements of study design early in the paper	Page 3
Setting	#5	Describe the setting, locations, and relevant dates, including periods of recruitment, exposure, follow-up, and data collection	Page 3
Eligibility criteria	#6a	Give the eligibility criteria, and the sources and methods of selection of participants.	Pgs 3,4&5
	#7	Clearly define all outcomes, exposures, predictors, potential confounders, and effect modifiers. Give diagnostic criteria, if applicable	Page 4&5
Data sources / measurement	#8	For each variable of interest give sources of data and details of methods of assessment (measurement). Describe comparability of assessment methods if there is more than one group. Give information separately for exposed and unexposed groups if applicable.	Pgs 5 & 6
Bias	#9	Describe any efforts to address potential sources of bias	Page 5&6

For peer review only

Study size	#10	Explain how the study size was arrived at	Page 4
Quantitative variables	#11	Explain how quantitative variables were handled in the analyses. If applicable, describe which groupings were chosen, and why	Pages 6, 7, 10,11 & 12.
Statistical methods	#12a	Describe all statistical methods, including those used to control for confounding	Pages 6 & 7
Statistical methods	#12b	Describe any methods used to examine subgroups and interactions	Pages 6, 7, 10,11 & 12.
Statistical methods	#12c	Explain how missing data were addressed	Page 6
Statistical methods	#12d	If applicable, describe analytical methods taking account of sampling strategy	Pages 6& 7
Statistical methods	#12e	Describe any sensitivity analyses	Pages 6, 7, 10,11 & 12.
**Results**			
Participants	#13a	Report numbers of individuals at each stage of study—eg numbers potentially eligible, examined for eligibility, confirmed eligible, included in the study, completing follow-up, and analysed. Give information separately for for exposed and unexposed groups if applicable.	Page 5

For peer review only

Participants	#13b	Give reasons for non-participation at each stage	Page 5
Participants	#13c	Consider use of a flow diagram	n/a
Descriptive data	#14a	Give characteristics of study participants (eg demographic, clinical, social) and information on exposures and potential confounders. Give information separately for exposed and unexposed groups if applicable.	Page 8
Descriptive data	#14b	Indicate number of participants with missing data for each variable of interest	n/a
Outcome data	#15	Report numbers of outcome events or summary measures. Give information separately for exposed and unexposed groups if applicable.	Page 8
Main results	#16a	Give unadjusted estimates and, if applicable, confounder-adjusted estimates and their precision (eg, 95% confidence interval). Make clear which confounders were adjusted for and why they were included	Pages 8 to 12
Main results	#16b	Report category boundaries when continuous variables were categorized	Pages 8 to 12
Main results	#16c	If relevant, consider translating estimates of relative risk into absolute risk for a meaningful time period	n/a
Other analyses	#17	Report other analyses done—e.g., analyses of subgroups and interactions, and sensitivity analyses	Pages 8 to 12

Discussion

For peer review only

Key results	#18	Summarise key results with reference to study objectives	Pages 12 to 15
Limitations	#19	Discuss limitations of the study, taking into account sources of potential bias or imprecision. Discuss both direction and magnitude of any potential bias.	Page ii and 15
Interpretation	#20	Give a cautious overall interpretation considering objectives, limitations, multiplicity of analyses, results from similar studies, and other relevant evidence.	Page 15
Generalisability	#21	Discuss the generalisability (external validity) of the study results	Page 15
**Other Information**			
Funding	#22	Give the source of funding and the role of the funders for the present study and, if applicable, for the original study on which the present article is based	Page ii

None The STROBE checklist is distributed under the terms of the Creative Commons Attribution License CC-BY. This checklist can be completed online using https://www.goodreports.org/, a tool made by the EQUATOR Network in collaboration with Penelope.ai

For peer review only

BMJ Open

Prevalence of Hepatitis B Virus infection and associated risk factors among pregnant women attending antenatal clinic in Mulago Hospital, Uganda: A cross-sectional study.

Journal:	*BMJ Open*
Manuscript ID	bmjopen-2019-033043.R2
Article Type:	Original research
Date Submitted by the Author:	28-Apr-2020
Complete List of Authors:	Kayondo, Simon Peter; Makerere University College of Health Sciences, Obstetrics and Gynaecology; Association of Obstetricians and Gynaecologists of Uganda,<br>Byamugisha, Josaphat; Makere University/Mulago National Referral Hospital, Obstetrics and Gynecology<br>Ntuyo, Peter; Makere University/Mulago National Referral Hospital, Obstetrics and Gynecology
**Primary Subject Heading**:	Obstetrics and gynaecology
Secondary Subject Heading:	Obstetrics and gynaecology, Infectious diseases, Haematology (incl blood transfusion)
Keywords:	Hepatitis B, prevalence, pregnant women, Mulago hospital, Uganda

SCHOLARONE™
Manuscripts

I, the Submitting Author has the right to grant and does grant on behalf of all authors of the Work (as defined in the below author licence), an exclusive licence and/or a non-exclusive licence for contributions from authors who are: i) UK Crown employees; ii) where BMJ has agreed a CC-BY licence shall apply, and/or iii) in accordance with the terms applicable for US Federal Government officers or employees acting as part of their official duties; on a worldwide, perpetual, irrevocable, royalty-free basis to BMJ Publishing Group Ltd ("BMJ") its licensees and where the relevant Journal is co-owned by BMJ to the co-owners of the Journal, to publish the Work in this journal and any other BMJ products and to exploit all rights, as set out in our licence.

The Submitting Author accepts and understands that any supply made under these terms is made by BMJ to the Submitting Author unless you are acting as an employee on behalf of your employer or a postgraduate student of an affiliated institution which is paying any applicable article publishing charge ("APC") for Open Access articles. Where the Submitting Author wishes to make the Work available on an Open Access basis (and intends to pay the relevant APC), the terms of reuse of such Open Access shall be governed by a Creative Commons licence – details of these licences and which Creative Commons licence will apply to this Work are set out in our licence referred to above.

Other than as permitted in any relevant BMJ Author's Self Archiving Policies, I confirm this Work has not been accepted for publication elsewhere, is not being considered for publication elsewhere and does not duplicate material already published. I confirm all authors consent to publication of this Work and authorise the granting of this licence.

For peer review only

Prevalence of Hepatitis B Virus infection and associated risk factors among pregnant women attending antenatal clinic in Mulago Hospital, Uganda: A cross-sectional study.

S.P. Kayondo[1], J. Byamugisha[2], P. Ntuyo[3].

Authors:

1. **Dr. Kayondo Simon Peter MBChB, M. Med Obs/Gyn (MUK)**
   Project Coordinator,
   Association of Obstetricians & Gynaecologists of Uganda (AOGU)
   P.O. Box 11966,
   Kampala- Uganda.
   Email: **kayondosimondr@gmail.com**
   Mobile: +256782736078

2. **Assoc. Prof. Josaphat Byamugisha, MBChB, M. Med Obs/Gyn, Ph.D. (MUK)**
   Associate Professor,
   Department of Obstetrics and Gynaecology,
   Makerere University College of Health Sciences,
   P.O. Box 7072**,**
   Kampala- Uganda**.**
   Email: **byamugisha2001@yahoo.com**
   Mobile: +256772580330

3. **Dr. Peter Ntuyo, MBChB, M. Med Obs/Gyn (MUK)**
   Obstetrician and Gynecologist,
   Directorate of Obstetrics and Gynecology,
   Mulago Hospital,
   P.O. Box 7051**,**
   Kampala- Uganda**.**
   Email: **peterntuyo@yahoo.com**
   Mobile: +256772481636.

Correspondence author:

Dr. Kayondo Simon Peter MBChB, M. Med Obs/Gyn (MUK)
Project Coordinator,
Association of Obstetricians & Gynaecologists of Uganda (AOGU)
P.O. Box 11966**,**
Kampala- Uganda**.**
Email: **kayondosimondr@gmail.com**
Mobile: +256782736078

For peer review only

ABSTRACT

Aim To determine the prevalence and factors associated with Hepatitis B Virus infection, among pregnant women attending antenatal clinic in Mulago Hospital.

Design Cross-sectional observational study.

Setting Mulago National Referral Hospital, Uganda, Antenatal Clinic.

Participants We randomly selected 340 pregnant women attending their first antenatal visit at Mulago Hospital Antenatal clinic**.**

Primary Outcome Hepatitis B surface antigen (HBsAg) positivity.

Results We recruited 340 participants, with a mean age of 27±5.7 years, and a median gravidity of 3. The prevalence of Hepatitis B Virus infection among pregnant women attending the antenatal clinic in Mulago Hospital, in our study, was 2.9% (95% CI, 1.58-5.40; n=10). Factors positively associated with Hepatitis B virus infection were: marital status, (aOR=11.37, p=0.002), having a Hepatitis B positive family member, (aOR=49.52, p<0.001), and having had a blood or body fluid splash to mucous membranes from a Hepatitis B positive patient, (aOR=61.69, p=0.015). Other factors such as age, socioeconomic status, number of sexual partners, HIV serostatus, piercing of ears, and history of blood transfusion, were not significantly associated with Hepatitis B virus infection in this study.

Conclusion The prevalence of Hepatitis B virus infection among pregnant women attending antenatal clinic in Mulago Hospital, was of intermediate endemicity. We found that marital status, having a Hepatitis B positive family member at home and having had a blood or body fluid splash to mucous membranes from a Hepatitis B positive patient, were independently associated with Hepatitis B infection. Factors such as age, HIV status, history of blood transfusion, piercing of ears, and social status were not associated with Hepatitis B status in this study.

keywords: Hepatitis B, prevalence, pregnant women, Mulago Hospital, Uganda.

For peer review only - http://bmjopen.bmj.com/site/about/guidelines.xhtml

Strengths and limitations of this study.

- We followed standard laboratory procedures, at an ISO 9001 certified laboratory using a highly sensitive, and specific HBsAg test kit performed by qualified personnel.
- Laboratory technologists performed all testing blinded through use of coded specimens.
- We recruited the study participants using a random sampling procedure.
- Our study setting was a national referral and teaching hospital: hence our findings may not be generally applicable in other settings.
- Because of limited number of positive cases in our study, we had limited power to assess the significance of associated factors.

Funding statement DELTAS Africa Initiative grant #DEL-15-011 to THRiVE-2 supported this work. The DELTAS Africa Initiative is an independent funding scheme of the African Academy of Sciences (AAS)'s Alliance for Accelerating Excellence in Science in Africa (AESA) and supported by the New Partnership for Africa's Development Planning and Coordinating Agency (NEPAD Agency) with funding from the Wellcome Trust grant #107742/Z/15/Z and the UK government. The views expressed in this publication are those of the authors and not necessarily those of AAS, NEPAD Agency, Wellcome Trust, or the UK government.

Conflict of interest: The authors declare no conflict of interest.

For peer review only

For peer review only - http://bmjopen.bmj.com/site/about/guidelines.xhtml

Introduction.

Hepatitis B is a viral infection of the liver that can cause acute and chronic disease. Worldwide, about two billion people have been exposed to hepatitis B, and about 257 million people are living with hepatitis B infection (defined as HBsAg positive) (1). Hepatitis B infection resulted in 887,000 deaths in 2015, mostly from liver complications: liver cirrhosis and hepatocellular carcinoma. Globally, hepatitis B prevalence in adults is highest in the WHO Western Pacific Region (6.2%), and the WHO African region (6.1%). In Sub-Saharan Africa, the overall HBsAg carrier rate in the general population is 5-20%, which is among the highest in the world. Perinatal transmission is estimated at 1-5% (2). 72% of the Ugandan population are exposed to hepatitis B in their lifetime, leading to a national prevalence of hepatitis B infection of approximately 10%. There is regional variation within Uganda however; the highest prevalence is in the northern part of the country, ranging from 19% in the North West to 25% in the North East (3, 4). It is not known how many pregnant women are hepatitis B positive worldwide. However, in Northern Uganda, prevalence is around 11.8% (5). Namirembe et al. estimated the prevalence among the antenatal population of Mulago Hospital at 0.9% in 2016 (6), although this study had several limitations with regard to participant recruitment and estimation of mother-to -child transmission risk.

In highly endemic areas, vertical transmission of hepatitis B from mother to child at birth plays a key role. However, horizontal transmission in childhood also occurs (1, 7). Infants infected from their mothers, or before the age of five, often develop chronic infection (1, 8, 9). Hepatitis B can also be transmitted through percutaneous or mucosal exposure to infected blood or body fluids, such as saliva, menstrual, vaginal, and seminal fluids (including in men who have sex with men) (1, 10). Re-use of needles and intravenous drug use are also high-risk activities for hepatitis B transmission. The infection may be transmitted through unsafe medical, dental, and surgical procedures, and through practices such as tattooing, or use of sharp instruments contaminated with blood (1).

The risk of developing chronic hepatitis B for babies born to a mother with hepatitis B is greater than 90%, if not identified and treated at birth. It is therefore imperative for pregnant women to know their hepatitis B status, ideally through testing in the first trimester (1). If the mother is hepatitis B positive, the baby should receive both the first dose of the hepatitis B vaccine and hepatitis B immunoglobulin at birth. This treatment is recommended by the CDC, although it may

For peer review only - http://bmjopen.bmj.com/site/about/guidelines.xhtml

not be available in all countries (1, 11, 12). If treatment is given promptly within the first 12 hours of life, the new-born has >90% chance of life-long protection against hepatitis B infection (11-13). Infant vaccination for hepatitis B was introduced in Uganda in 2002 (4), as part of the Uganda National Expanded Programme for Immunisation (UNEPI) schedule. However, adults born prior to 2002 (currently aged >18 years) remain a high-risk group.

Acute or chronic hepatitis B infection in pregnancy, is generally similar to that in the general population. Hepatitis B in pregnancy is not teratogenic and does not increase maternal or fetal mortality (14, 15). However acute infection is associated with a higher incidence of low birth weight and prematurity than in the general population (15). In pregnancy, the immune response against hepatitis B is less effective, possibly due to a shift from the Th1 to Th2 response. The WHO defines hepatitis B infection in newborns as HBsAg positivity 6 months after birth (1, 16). The risk of mother-to-child transmission is very high in the absence of prophylaxis, varying with the HBeAg/anti-HBe status of the mother: 70-90% for HBeAg positive, 25% for HBeAg negative/HBeAb-negative mothers and 12% for HBeAg-negative/anti HBe positive mothers (13, 15, 17).

We aimed to determine the prevalence of and risk factors associated with hepatitis B infection among pregnant women attending for antenatal care at Mulago Hospital in Kampala, Uganda.

Methods:

Study design and setting.

This was a cross-sectional study, conducted at Mulago Hospital antenatal clinic. Mulago Hospital was founded in 1917 in Kampala City, the capital of Uganda, and is the Ugandan national referral hospital. It is a public general and teaching hospital, with a bed capacity of over 1500 beds. Mulago Hospital is located on Mulago hill, approximately 5km northeast of Kampala's central business district. Approximately 2500 women attend for antenatal care at Mulago Hospital every month.

.

We purposively selected Mulago Hospital because it serves people from all ethnic groups and regions of Uganda. It also serves a high number of refugees, such as those from Rwanda, South Sudan and Congo.

For peer review only - http://bmjopen.bmj.com/site/about/guidelines.xhtml

Kawempe General Hospital, in Kawempe Division, temporarily houses the Mulago Hospital antenatal clinic, as the main Mulago Hospital is undergoing major renovation. The clinic continues to offer preventive and curative services, such as health education, immunization, prenatal maternal and fetal monitoring. Between 200 and 250 pregnant women visit the clinic daily on Tuesday, Wednesday and Thursday, of whom about 100 each day are attending their first antenatal visit. We enrolled only mothers attending their first visit into the study, as the flow of first visit attendees in the ANC clinic was convenient for recruitment, unlike their repeat visit counterparts. We enrolled women after their health education session, as they awaited examination.

Study population.

The study population was pregnant women attending antenatal clinic, between December 2018 and February 2019, in Mulago Hospital, Kawempe site.

Eligibility criteria.

Inclusion criteria:

All pregnant women who were attending their first antenatal visit at Mulago Hospital who were willing to participate in the study. Women below 18 years were eligible for recruitment as emancipated minors.

Exclusion criteria:

We excluded women who could not understand either English or Luganda, which were the two languages used for the consenting process and administration of the questionnaires.

Research variables:

The dependent variable was hepatitis B infection among pregnant women, assessed by HBsAg positivity.

The independent variables were factors associated with positive hepatitis B status. These included the sociodemographic factors such as age, marital status, level of education, socio-economic status, and sexual behavior. We also included obstetric factors, such as gravidity and parity, and other factors such as history of blood transfusion, history of caring for a hepatitis B positive patient, immunization status, ear piercings, tattoos, and intravenous drug use in our model.

Sample size estimation.

We estimated the sample size for prevalence using the Kish and Leslie formula, 1965 (18).

For peer review only - http://bmjopen.bmj.com/site/about/guidelines.xhtml

Using a prevalence from Northern Uganda of 11.8% (5), the required sample size to determine prevalence would be 160 women.

We based the sample size estimates for factors associated with hepatitis B infection in pregnancy on the hypothesis that the prevalence of infection would be higher among women ≤20 years than among those >20 years, as found by Bayo et al. (5). We compared the proportions in sub-populations (REF: Fleiss, Statistical Methods for Rates and Proportions, formulas pages 3.18 & 3.19) using an OpenEpi Calculator (accessed at **http://www.openepi.com/SampleSize/SSCC.htm).** Assuming power of 80%, type 1 error of 5% and frequency of hepatitis B infection of 20% among women ≤20 years and 8.7% among women >20 years (5), we calculated a total sample size required for risk factor modelling of 340. 340 women were included in the study.

Sampling procedure.

We used a random probability sampling method. During the day of data collection, we used the register book for the antenatal clinic attendance and the booking cards for the first visit attendees to identify eligible potential participants. We invited every 10th eligible woman to take part in the study. If the 10th woman declined participation in the study, then the next eligible woman was invited. There was an average daily attendance of ~100 eligible potential participants on three days per week. Data were collected over a three-month period (December 2018 and February 2019).

Study Procedure.

The antenatal clinic midwives, who were the research assistants, approached potential participants, and explained the study purpose to them. They then screened them for eligibility and obtained informed consent for participation in the study. We used interviewer-administered semi-structured questionnaires to get socio-demographic information including age, gestational age, gravidity, parity, education and occupation, and other factors that could influence infection with HBV, such as injection drug use, tattoos, family history of hepatitis B infection, sexual history, among others. Obstetric factors like gravidity and parity, previous history of taking care of a hepatitis B positive patient, immunization against hepatitis B, were all assessed.

The laboratory technician collected four millilitres (4mls) of whole blood from the antecubital fossa under aseptic technique, into BD Clot Activator Tube vacutainers (labelled with the participant's study number), using WHO (2010) guidelines on blood collection (19). The

For peer review only - http://bmjopen.bmj.com/site/about/guidelines.xhtml

technician then took the blood to the Kawempe Hospital Laboratory, centrifuged, and transferred an aliquot of serum, about 2mls, into cryovials. We performed immunochromatographic assay using HBsAg Rapid Test Strip, FaStep, for HBsAg testing following the manufacturer's instructions. This rapid test kit has a relative sensitivity of >99.9%, a relative specificity of 99.9%, and an accuracy of >99.9%. We did quality control on this test kit using known positive and negative samples from MBN Clinical Laboratories, an ISO 9001 certified laboratory, before it was used to test any participant's samples. We performed the HBsAg rapid tests using the following procedure: The laboratory technician labelled a rapid test strip with the patient identifiers, and placed it on a flat surface. He pipetted 3 drops of serum from the cryovial and applied it to the sample pad of the test strip. We read the result after 15 minutes. A positive result was one where two-coloured bands appeared on the membrane, one in the control region, and another in the test region. A negative result was one, where only one band appeared in the control region, and none in the test region. An invalid result was one where a band failed to appear in the control region.

We produced results and gave a copy to the patient after being counselled by the principal investigator and the nurse counsellor. We sent all HBsAg positive samples to MBN Clinical Laboratories, Kampala, an ISO 9001 accredited laboratory, for confirmatory HBsAg testing, HBeAg testing, and liver function tests. We took 5% of all HBsAg negative samples to MBN, for external quality assurance. We packed and transported all samples at 2-8 degrees Celsius, on the same day of collection. We referred hepatitis B positive mothers to the hepatitis B Clinic in Mulago Hospital (Kiruddu) for further care, where the physicians performed their viral load assays and further assessments for treatment. We planned to vaccinate their babies against hepatitis B within 24 hours of birth. The study endeavoured to meet the cost of the HepB-BD vaccine for all study participants who were hepatitis B positive.

Data Management and Analysis.

The principal investigator double-entered the raw study data into Epidata 3.1 and imported into STATA 14.1. Numerical results are presented as frequencies or percentages. To assess factors associated with hepatitis B infection in pregnancy, we performed bivariate analysis and computed crude odds ratios and 95% confidence intervals. Student's t-test was used to assess statistical significance between groups for continuous variables, while the Fischer's exact test was used for categorical variables.

For peer review only - http://bmjopen.bmj.com/site/about/guidelines.xhtml

To assess the independent association of these risk factors, we performed multivariate analysis. All independent variables with a p-value of less than 0.2 at bivariate analysis and those with biological significance were included in multivariate logistic regression. Stepwise regression method was used to determine the model of best fit. We considered all associations with p-value <0.05 as statistically significant.

Ethical considerations.

Ethical approval was granted by the Department of Obstetrics and Gynaecology, Makerere University, and Makerere University School of Medicine Research and Ethics Committee, **(#REC REF 2018-119),** Mulago Hospital Research and Ethics Committee, **(#MHREC1519**), and the Uganda National Council for Science and Technology, **(#HS257ES),** before any collection of samples or data.

We explained information regarding the study to the subjects and got written consent for participation in the study in English/Luganda. We interviewed participants and collected samples, following local guidelines regarding research participant privacy. Only study numbers, with unique participant identifiers, were used to label the questionnaires and the samples. We kept filled questionnaires separate from signed consent forms and locked away securely all study material, with only access to study team. Participants were free to drop out at any point of the study, with an assurance that if they did so this would not affect their ongoing antenatal care.

All researchers in the study were fully immunized against hepatitis B. They wore protective gear, such as gloves, masks, and eye glasses during laboratory procedures. These measures protected both mothers and health workers from acquiring hepatitis B infection from each other during the study.

Patient and Public involvement While there were patients involved in this study as study participants, we did not involve them at the stage of the study design. However, the intended outcome in favour of the patient was that we made available Hepatitis B birth dose vaccination for all exposed babies. We circulated a summary of the findings using SMS messaging to all study participants.

For peer review only - http://bmjopen.bmj.com/site/about/guidelines.xhtml

Results.

Description of the study population.

We recruited 340 participants into the study, with a mean age of 27±5.7 years (Table 1). 56.8% were from Kampala, 40% from Wakiso, and 3.2% from elsewhere**.** Most of the participants (76.7%) had a secondary education and above, and 52.4% were employed. A little over a half of the participants (51.7%) were business people, 41.5% in professional jobs, 5.1% casual laborers, while 3% were health workers. More than half (65.3%), were of low socioeconomic status, earning less than 500,000 Uganda shillings per month. The majority of the participants (70.3%) had their sexual debut during their teenage years.

Table 1: Table showing demographic/gynecological/obstetric/sexual characteristics of the study participants. Mean (S.D.) for numerical data, n (%) for categorical data.

Demographic Characteristics	Frequency [n=340, (%)]
**Age (years); mean (std. dev)**	27 (5.7)
**Age (years) categorized.**	
≤20	28 (8.2)
>20	312 (91.8)
**Employment status.**	
Yes	178 (52.4)
No	162 (47.6)
**Marital status.**	
Living with a partner.	304 (89.4)
No stable partner.	36 (10.6)
**Gyn/Obs/Sexual characteristics.**	
**Gravidity; median (IQR)**	3 (2 - 3)
**Gravidity categorized.**	
1	67 (19.7)
2-4	211 (62.1)
>4	62 (18.2)
**Weeks of amenorrhea (WOA) categorized.**	
>20	211 (62.1)
≤20	54 (15.9)
Unknown	75 (22.1)
**Mother Hepatitis B positive?**	
Yes	4 (1.2)
No	126 (37.1)
I don’t know.	210 (61.7)
**Number of sex partners; median (IQR)**	2 (1-3)
**Number of sex partners categorized.**	
1	117 (34.6)
≥2	221 (65.4)

Over half of the participants were employed, the majority (89.4%) were living with a partner, and most of them had more than one lifetime sexual partner (Table 1).

For peer review only - http://bmjopen.bmj.com/site/about/guidelines.xhtml

Factors associated with Hepatitis B virus infection.

Table 2: Table showing immunosuppression characteristics, vaccination history, familial exposure, occupational exposure risk of study participants, and exposure to Hepatitis B through sharp instruments, medical and surgical procedures.

	Freq [N=340, (%)]
**Immunosuppression characteristics.**	
**HIV sero-status**	
Positive	10 (2.9)
**History of Diabetes mellitus.**	
Yes	3 (0.9)
No	47 (13.8)
Unknown	290 (85.3)
**Vaccination history.**	
**Hepatitis B vaccination in the first year of life.**	
Yes	9 (2.6)
No	240 (70.6)
Don't know.	91 (26.8)
**Hepatitis B vaccination after the age of 10 years.**	
Yes	12 (3.5)
No	299 (88.0)
Don't know.	29 (8.5)
**Exposure through sharp instruments/medical/surgical procedures.**	
**Ever had a blood transfusion.**	
Yes	18 (5.3)
**Ever pierced your ears.**	
Yes	275 (80.9)
**Familial exposure**	
**Have a Hepatitis B positive family member at home?**	
Yes	7 (2.1)
No	291 (85.6)
Don't know.	42 (12.3)
**Occupational exposure.**	
**Ever had needle stick injury?**	
Yes	4 (1.2)
**Blood or body fluid splash from Hepatitis B positive patient.**	
Yes	2 (0.6)

Only 2.9% of study participants were HIV positive (Table 2). Vaccination against hepatitis B was low, with 2.6%, and 3.5% as the childhood and adult vaccination rates, respectively (Table 2). About 2.1% of the participants reported having a hepatitis B positive family member at home (Table 2).

For peer review only

For peer review only - http://bmjopen.bmj.com/site/about/guidelines.xhtml

Prevalence

10 out of 340 participants were HBsAg positive, comprising 2.9% (95% CI, 1.58-5.40).

All the HBsAg positive participants were HBeAg negative and had normal liver function tests.

Associations between the independent variables and Hepatitis B status.

Table 3: Table showing association between demographic, gynecological, obstetric characteristics and Hepatitis B status.

Demographic characteristics.	Hepatitis B status.		Unadj Odds (95% CI)	P-value.
	Pos [n=10, (%)]	Neg [n=330, (%)]		
**Age (years); mean (std. dev)**	27.4 (6.2)	26.9 (5.7)		**0.775[a]**
**Age (years) categorised.**				**0.582**
<20	1 (10.0)	27 (8.2)	1.00	
≥20	9 (90.0)	303 (91.8)	1.25 (0.15 - 10.21)	
**Employment status.**				**0.753**
Yes	6(60)	172(52.2)	1.00	
No	4(40)	158(47.8)	0.73(0.20-2.62)	
**Marital status.**				**0.004**
Staying with a partner.	6 (60.0)	298 (90.3)	1.00	**0.014**
No stable partner.	4 (40.0)	32 (9.7)	6.21 (1.66 - 23.16)	
**Gynecological/Obstetric factors.**				
**WOA categorized.**				**<0.001**
>20	1 (10.0)	210 (63.6)	1.00	
≤20	6 (60.0)	48 (14.6)	26.25 (3.09 - 223.13)	
Unknown	3 (30.0)	72 (21.8)	8.75 (0.90 - 85.46)	
**Mother Hepatitis B positive?**				**0.036**
Yes	1 (10.0)	3 (0.9)	1.00	
No	1 (10.0)	125 (37.9)	0.02 (0.00 - 0.48)	
I don't know.	8 (80.0)	202 (61.2)	0.12 (0.01 - 1.27)	

P- Fishers exact p-values, P[a]- t-test p-values.

In bivariate analysis, we found the participant's marital status, weeks of amenorrhea, mother's hepatitis B status, significantly associated with hepatitis B status (Table 3). These were included as independent variables in multivariable analysis together with age of the woman.

For peer review only - http://bmjopen.bmj.com/site/about/guidelines.xhtml

For peer review only

Table 4: Association of immunosuppression characteristics/occupational exposure risk/surgical procedures/sharp instruments and familial exposure with Hepatitis B status.

**Immunosuppression characteristics.**	**Hepatitis B Status.**		**Unadjusted Odds (95% CI)**	**P-value.**
	**Pos [n=10, (%)]**	**Neg [n=330, (%)]**		
**HIV sero-status**				**0.261**
Positive	1 (10.0)	9 (2.7)	3.96 (0.45—34.69)	
Negative	9 (90.0)	321 (97.3)	1.00	
**Occupational exposure risk.**				
**Blood or body fluid splash from a Hepatitis B positive patient.**				**0.058**
Yes	1(10)	1(0.3)	36.56 (2.11—631.85)	
No	9(90)	329(99.7)	1.00	
**Exposure through surgical procedures/sharp instruments.**				
**Ever had a blood transfusion.**				**0.424**
Yes	1 (10.0)	17 (5.2)	2.05 (0.24—17.09)	
No	9 (90.0)	313 (94.8)	1.00	
**Ever pierced your ears.**				**0.411**
Yes	7 (70.0)	268 (81.2)	1.00	
No	3 (30.0)	62 (18.8)	1.85 (0.47—7.37)	
**Familial exposure**				
**Have a Hepatitis B positive family member at home?**				**0.011**
Yes	2 (20.0)	5 (1.5)	1.00	
No	6 (60.0)	285 (86.4)	19.00 (3.05 - 118.24)	
Don't know.	2 (20.0)	40 (12.1)	2.38 (0.46 – 12.17)	

Having had a blood or body fluid splash to the mucous membranes from a Hepatitis B positive patient, and having a Hepatitis B positive family member were significantly associated with Hepatitis B status, at bivariate analysis (Table 4). Both were included in multivariable analysis.

For peer review only - http://bmjopen.bmj.com/site/about/guidelines.xhtml

Table 5: Multivariable logistic regression model showing factors associated with Hepatitis B virus infection among study participants.

Characteristics	Adjusted Odds ratio (95% CI)	P-value
**Age(years) categorized**		
<20	1.00	
≥20	0.27 (0.01 - 5.31)	**0.387**
**Marital status**		
Staying with a partner	1.00	
No stable partner	11.37 (2.37 - 54.60)	**0.002**
**Have a Hepatitis B positive family member at home**		
No	1.00	
Yes	49.52 (6.24 - 392.85)	**<0.001**
Don't know.	4.25 (0.71 - 25.35)	**0.113**
**Blood or body fluid splash from Hepatitis B positive patient**		
No	1.00	
Yes	61.69 (2.24 - 1701.54)	**0.015**

Marital status, having a hepatitis B positive family member, and history of a blood or body splash to mucous membranes from a hepatitis B positive patient were all significantly independently associated with positive testing for hepatitis B (Table 5). The association with age was not statistically significant (Table 5).

Data availability statement: We have included all data relevant to this study in the article or uploaded them as supplementary information. Raw data are accessible on: https://drive.google.com/drive/folders/1wc0C3bP4Qge0OKtG4z-zXC6pbD1F3nsL?usp=sharing.

Discussion

We conducted this study to determine the prevalence and risk factors associated with hepatitis B infection among pregnant women attending the antenatal clinic in Mulago Hospital, a multicultural, multiracial national referral hospital in urban Uganda.

Although Namirembe et al., in early 2016, estimated the prevalence of hepatitis B among pregnant women at 0.9%, before Mulago Hospital was transferred to the new Kawempe site (6), their study was not without limitations. Notably, the prevalence of hepatitis B in their cohort was much lower, compared to our cohort (0.9% v. 2.9%). Apart from having a different socio-demographic profile, the 2016 study excluded pregnant women below 14 years, and above 43 years. It also excluded all participants who were bedridden, and all those with obstetric

For peer review only - http://bmjopen.bmj.com/site/about/guidelines.xhtml

emergencies. Application of these exclusion criteria may have skewed the participating population compared to the general obstetric population. A further explanation for the observed difference may be because Kawempe, the site at which we performed our study, is an area of significant socio-economic deprivation, where high-risk activities such as prostitution are common. By contrast the 2016 study was performed at the old Mulago site, where the risk profile of the population is likely to be less. Nonetheless, since majority (60%) of all hepatitis B positive pregnant women present in the first half of the pregnancy, as shown by our study (Table 3), it is an opportunity for early testing and intervention to reduce mother-to-child transmission.

The prevalence of hepatitis B in our study (2.9%) falls with the WHO 'intermediate endemicity' category (2-8%) (20). It is comparable to the prevalence previously reported by Pirillo et al. in Kampala (4.9%). Internationally, the prevalence in our study is also similar to two Rwandan studies that reported 2.4% and 3.7% respectively (21, 22). However, the Rwandan studies were both conducted in HIV positive pregnant women only. Other studies from Kenya and Ethiopia respectively reported prevalences of hepatitis B among pregnant women attending antenatal care of 3.8%, and 3.5% respectively (23,24). These prevalences are also comparable to our study. By contrast, low endemicity areas, such as the United States of America, report values around 0.38% (25). These low endemicity areas have vigorously implemented hepatitis B prevention strategies, such as universal testing of all pregnant women for hepatitis B, universal vaccination at birth, and universal childhood immunization. These strategies are uncommon in intermediate and high endemicity areas (26).

The 11.8% prevalence of hepatitis B in pregnant women found by Bayo et al. in Northern Uganda in 2014(5), and the average national prevalence of ~10% in the general population (4), are both higher than the prevalence in our study. The prevalence found in other select populations such as health workers in Mulago Hospital is also higher than in our study (8.1% (27)). Several factors may account for this difference. The Central region of Uganda is a low prevalence area, compared to other regions such as the North and East. The intensification of hepatitis B prevention programmes over the last few years in Uganda may also have contributed to a reduction in prevalence of hepatitis B in the general population and among pregnant women.

In our study, we found marital status, history of having had a hepatitis B positive family member, and history of having had a blood or body fluid splash from a hepatitis B positive patient, as factors independently associated with hepatitis B infection (Table 5). Other factors identified as significant in previous studies, such as age (5, 25, 28, 29), level of education, (29, 30) socioeconomic status (31), number of sexual partners (24, 32), piercing of ears (32), history of abortions (23, 24), history of blood transfusion (28), and history of jaundice (33), were not significantly associated with hepatitis B infection in this study. HIV serostatus, a factor that several studies have found associated with hepatitis B infection (28, 31), did not have statistical significance in our study (Table 4). This could be because the HIV prevalence in our study was generally low (Table 2).

For peer review only - http://bmjopen.bmj.com/site/about/guidelines.xhtml

Women who did not have a stable partner, were eleven (11) times as likely to be Hepatitis B positive, as their counterparts who lived with partners. (aOR=11.37, 95% CI, (2.37-54.60), p= 0.002, Table 5). Similar results have been reported from Juba, South Sudan, although the increased risk was specific to loss of a partner; (OR 4.4, CI 1.4 -13.9) (33). Chernet et al., and Umare et al. also found an association between multiple sexual partners and hepatitis B positivity among pregnant women in Ethiopia, (aOR=6.923 and aOR=16.8, respectively) (24, 32). By contrast, studies from Kenya and Nigeria did not find any significant association between marital status and hepatitis B status, (23, 34). In our study, the women with no stable partner were widowed, separated, or unmarried. This increases the risk of having contracted hepatitis B from the lost partner or via risk-taking sexual behaviour.

Study participants who had a hepatitis B positive family member, were almost fifty (50) times more likely to be positive for hepatitis B, than those who did not (aOR=49.52, 95% CI: 6.24-392.85, p<0.001, Table 5). Data from the CDC supports this finding, showing that 3-20% of close contacts of people with chronic hepatitis B are also infected. Screening and vaccination of all close contacts of known hepatitis B cases is therefore recommended (35). A study from South Sudan also showed an increased risk of hepatitis B infection among pregnant women who reported a history of loss of a partner (OR=4.4) (33). This important finding underscores the risk of horizontal transmission of hepatitis B infection, either sexually between partners, or through contact with blood and body fluids among people staying in the same home. This can be through sharing household items such as toothbrushes, razors, syringes, and needles. The risk posed by having to take care of a hepatitis B positive patient is even higher, with exposure to blood and body fluids infected with the virus. It is essential that those who live with, or take care of, a hepatitis B positive individual be fully vaccinated to reduce the risk. All hepatitis B positive mothers should be identified during antenatal care and vertical transmission prevented through hepatitis B vaccination of the infant within the first 24 hours after delivery.

Those who had ever had a blood or body fluid splash to mucous membranes from a hepatitis B positive patient, were almost 62 times more likely to be hepatitis B positive than those who were unexposed (aOR=61.69, 95% CI: 2.24-1701.54, p=0.015, table 5). This is a significant potential sources of hepatitis B infection, either from infected patient to a health-care worker or vice versa. A study by Ziraba et al. has previously shown that only small percentage of the health workers in our study setting were vaccinated (27). The WHO estimates the risk of infection with hepatitis B after a single percutaneous exposure to be 6-60%, which is higher than hepatitis C (2%) or HIV (0.1-0.3%) (36). It is extremely important to encourage vaccination of health-care workers and people in contact with hepatitis B- positive patients (37).

A potential limitation of our study is that we performed it in a national referral and teaching hospital, meaning it may not accurately reflect the situation in other settings. The limited number

For peer review only - http://bmjopen.bmj.com/site/about/guidelines.xhtml

of hepatitis B positive patients in our study also leads to increased uncertainty regarding the magnitude of risk conferred by each factor studied.

Conclusion

The prevalence of hepatitis B infection among pregnant women attending antenatal care clinic in Mulago Hospital is of intermediate endemicity. We found that several independent risk factors were associated with increased likelihood of being hepatitis B positive: marital status, history of having had a hepatitis B positive family member, and history of a blood or body fluid splash to mucous membranes from a hepatitis B positive individual. Factors such as age, HIV status, history of blood transfusion, piercing of ears, and social status were not associated with the likelihood of being hepatitis B positive.

Recommendations

Because hepatitis B is of intermediate endemicity in our population, we recommend testing all pregnant women for hepatitis B during antenatal care. All babies born to hepatitis B positive mothers should undergo the full schedule of hepatitis B vaccination, with or without hepatitis B immunoglobulin starting at birth.

Since most of the adult population of Uganda were not vaccinated in childhood, we recommend screening and adult vaccination for high-risk groups, such as health-care workers and people living or taking care of Hepatitis B positive individuals to reduce the likelihood of horizontal transmission.

We further recommend vaccination of pregnant women against hepatitis B, which is not contraindicated in pregnancy. There should be clear referral pathways for pregnant women who are hepatitis B positive to access specialist care for further serological, virologic and hepatological assessment and management, including long term monitoring.

Finally, we recommend a larger multicentre study in Uganda that includes tests for viral load and anti-hepatitis B core antibody. This will yield more important information regarding important exposures, risk of vertical transmission, and more generalizable risk factors.

Authors' contributions.

KSP developed the study's concept, designed the study methods, and also took part in data collection, analysis and interpretation. PN revised the manuscript critically and made amendments for important intellectual content; JB contributed significantly to the intellectual content of the manuscript and also gave the final approval of the version submitted. All the authors have agreed to be accountable for all aspects of the work in this study.

Authors' information.

For peer review only - http://bmjopen.bmj.com/site/about/guidelines.xhtml

KSP is an Obstetrician and Gynaecologist and Project Coordinator, Association of Obstetricians and Gynaecologists of Uganda (AOGU). PN is an Obstetrician and Gynaecologist, Directorate of Obstetrics and Gynaecology, Mulago Hospital. JB is an Associate Professor, Department of Obstetrics and Gynaecology, Makerere University College of Health Sciences.

Acknowledgements

We thank all senior members of the Department of Obstetrics and Gynaecology, Makerere University and members of the Directorate of Obstetrics and Gynaecology, Mulago Hospital, for the support and direction during the study. We thank Assoc. Prof. Annettee Nakimuli, for her tremendous input to this study and providing a conducive environment for us to carry it out. Great thanks to Mr. Godwin Anguzu, who offered technical input during data analysis. Finally, great thanks to the whole dedicated data collection team in the Mulago Hospital antenatal care clinic, Kawempe site.

REFERENCES

1. Organisation WHO. Hepatitis B Fact Sheet World Health Organisation; 2018 [

2. Kiire C, Group ARS. Hepatitis B infection in sub-Saharan Africa. Vaccine. 1990;8:S107-S12.
3. Ochola E, Ocama P, Orach CG, Nankinga ZK, Kalyango JN, McFarland W, et al. High burden of hepatitis B infection in Northern Uganda: results of a population-based survey. BMC Public Health. 2013;13(1):727.
4. Bwogi J, Braka F, Makumbi I, Mishra V, Bakamutumaho B, Nanyunja M, et al. Hepatitis B infection is highly endemic in Uganda: findings from a national serosurvey. African health sciences. 2009;9(2).
5. Bayo P, Ochola E, Oleo C, Mwaka AD. High prevalence of hepatitis B virus infection among pregnant women attending antenatal care: a cross-sectional study in two hospitals in northern Uganda. BMJ Open. 2014;4(11):e005889.
6. Allen N, Bashir M, Mugisha TI. Prevalence and Associated Factors of Hepatitis B Virus Infection among Pregnant Women Attending Antenatal Care Clinic at Mulago National Referral Hospital, Uganda. International Blood Research & Reviews. 2017:1-10.
7. Paul T, Marie TP, Bechem E. Knowledge, attitude and practice of staff of 4 hospitals in Yaoundé on the prevention of vertical transmission of hepatitis B. Pan African Medical Journal. 2017;28(1).
8. Ifeorah I, Bakarey A, Adewumi M, Faleye T, Akere A, Omoruyi C, et al. Patterns of serologic markers of hepatitis B virus infection and the risk of transmission among pregnant women in Southwestern Nigeria. Journal of Immunoassay and Immunochemistry. 2017;38(6):639-51.
9. Shi Y-H, Shi C-H. Molecular characteristics and stages of chronic hepatitis B virus infection. World journal of gastroenterology: WJG. 2009;15(25):3099.
10. Bodo B, Malande OO. Delayed introduction of the birth dose of Hepatitis B vaccine in EPI programs in East Africa: a missed opportunity for combating vertical transmission of Hepatitis B. The Pan African Medical Journal. 2017;27(Suppl 3).
11. Prevention CfDCa. Viral hepatitis, perinatal transmission 2018 [
12. Schillie S, Vellozzi C, Reingold A, Harris A, Haber P, Ward JW, et al. Prevention of hepatitis B virus infection in the United States: recommendations of the Advisory Committee on Immunization Practices. MMWR Recommendations and Reports. 2018;67(1):1.

For peer review only - http://bmjopen.bmj.com/site/about/guidelines.xhtml

13. Shimakawa Y, Seck A, Nayagam S, Toure-Kane C, Lemoine M. Screening strategies to prevent mother-to-child transmission of hepatitis B in sub-Saharan Africa. The Lancet Gastroenterology & Hepatology. 2018;3(4):222-3.
14. Benhammou V, Tubiana R, Matheron S, Sellier P, Mandelbrot L, Chenadec JL, et al. HBV or HCV Coinfection in HIV-1-Infected Pregnant Women in France: Prevalence and Pregnancy Outcomes. Journal of acquired immune deficiency syndromes (1999). 2018;77(5):439-50.
15. Borgia G, Carleo MA, Gaeta GB, Gentile I. Hepatitis B in pregnancy. World Journal of Gastroenterology: WJG. 2012;18(34):4677.
16. Organization WHO. Guidelines for the Prevention Care and Treatment of Persons with Chronic Hepatitis B Infection: Mar-15: World Health Organization; 2015.
17. Babatunde O, Smuts H, Eley B, Korsman S, De Lacy R, Hardie D. Fulminant hepatitis B virus (HBV) infection in an infant following mother-to-child transmission of an e-minus HBV mutant: Time to relook at HBV prophylaxis in South African infants. South African Medical Journal. 2018;108(5):389-92.
18. Israel GD. Determining sample size. 1992.
19. Organization WH. WHO guidelines on drawing blood: best practices in phlebotomy. 2010.
20. Zampino R, Boemio A, Sagnelli C, Alessio L, Adinolfi LE, Sagnelli E, et al. Hepatitis B virus burden in developing countries. World journal of gastroenterology. 2015;21(42):11941.
21. Pirillo MF, Bassani L, Germinario EA, Mancini MG, Vyankandondera J, Okong P, et al. Seroprevalence of hepatitis B and C viruses among HIV-infected pregnant women in Uganda and Rwanda. Journal of medical virology. 2007;79(12):1797-801.
22. Mutagoma M, Balisanga H, Malamba SS, Sebuhoro D, Remera E, Riedel DJ, et al. Hepatitis B virus and HIV co-infection among pregnant women in Rwanda. BMC infectious diseases. 2017;17(1):618.
23. Ngaira JAM, Kimotho J, Mirigi I, Osman S. Prevalence, awareness and risk factors associated with Hepatitis B infection among pregnant women attending the antenatal clinic at Mbagathi District Hospital in Nairobi, Kenya. The Pan African Medical Journal. 2016;24.
24. Chernet A, Yesuf A, Alagaw A. Seroprevalence of Hepatitis B virus surface antigen and factors associated among pregnant women in Dawuro zone, SNNPR, Southwest Ethiopia: a cross sectional study. BMC research notes. 2017;10(1):418.
25. Walker TY, Smith EA, Fenlon N, Lazaroff JE, Dusek C, Fineis P, et al. Characteristics of Pregnant Women With Hepatitis B Virus Infection in 5 US Public Health Jurisdictions, 2008-2012. Public Health Reports. 2016;131(5):685-94.
26. Visser M, van der Ploeg C, Smit C, Abbink F, Hukkelhoven C, Ommen P, et al. Evaluating progress towards triple elimination of mother-to-child transmission of HIV, syphilis and hepatitis B in the Netherlands, 2012-2015. 2018.
27. Abdhalah K Ziraba JB, Alice Namale, Caroline W Wainaina and Harriet Mayanja-Kizza. Sero-prevalence and risk factors for hepatitis B virus infection among health care workers in a tertiary hospital in Uganda. BMC Infectious Diseases. 2010;10(191).
28. Noubiap JJN, Nansseu JRN, Ndoula ST, Bigna JJR, Jingi AM, Fokom-Domgue J. Prevalence, infectivity and correlates of hepatitis B virus infection among pregnant women in a rural district of the Far North Region of Cameroon. BMC Public Health. 2015;15(1):454.
29. Souza MT, de Pinho TLR, Santos MDC, dos Santos A, Monteiro VL, Fonsêca LMB, et al. Prevalence of hepatitis B among pregnant women assisted at the public maternity hospitals of São Luís, Maranhão, Brazil. The Brazilian Journal of Infectious Diseases. 2012;16(6):517-20.
30. Utoo B. Hepatitis B surface antigenemia (HBsAg) among pregnant women in southern Nigeria. African health sciences. 2013;13(4):1139-43.
31. Manyahi J, Msigwa Y, Mhimbira F, Majigo M. High sero-prevalence of hepatitis B virus and human immunodeficiency virus infections among pregnant women attending antenatal clinic at Temeke

For peer review only - http://bmjopen.bmj.com/site/about/guidelines.xhtml

municipal health facilities, Dar es salaam, Tanzania: a cross sectional study. BMC pregnancy and childbirth. 2017;17(1):109.
32. Umare A, Seyoum B, Gobena T, Mariyam TH. Hepatitis B virus infections and associated factors among pregnant women attending antenatal Care Clinic at Deder Hospital, eastern Ethiopia. PloS one. 2016;11(11):e0166936.
33. Kirbak ALS. Sero-prevalence for Hepatitis B virus among pregnant women attending antenatal clinic in Juba Teaching Hospital, Republic of South Sudan. The Pan African medical journal. 2017;26.
34. Buseri F, Seiyaboh E, Jeremiah Z. Surveying infections among pregnant women in the Niger Delta, Nigeria. Journal of Global infectious diseases. 2010;2(3):203.
35. Weinbaum CM, Williams I, Mast EE, Wang SA, Finelli L, Wasley A, et al. Recommendations for identification and public health management of persons with chronic hepatitis B virus infection. 2008.
36. WHO. Exposure to blood or other body fluids

2009 [
37. Organization WHO. Hepatitis B vaccines: WHO position paper, July 2017–Recommendations. Vaccine. 2019;37(2):223-5.

For peer review only

For peer review only - http://bmjopen.bmj.com/site/about/guidelines.xhtml

Reporting checklist for cross sectional study.

Based on the STROBE cross sectional guidelines.

Instructions to authors

Complete this checklist by entering the page numbers from your manuscript where readers will find each of the items listed below.

Your article may not currently address all the items on the checklist. Please modify your text to include the missing information. If you are certain that an item does not apply, please write "n/a" and provide a short explanation.

Upload your completed checklist as an extra file when you submit to a journal.

In your methods section, say that you used the STROBE cross sectionalreporting guidelines, and cite them as:

von Elm E, Altman DG, Egger M, Pocock SJ, Gotzsche PC, Vandenbroucke JP. The Strengthening the Reporting of Observational Studies in Epidemiology (STROBE) Statement: guidelines for reporting observational studies.

For peer review only

		Reporting Item	Page Number
**Title and abstract**			
Title	#1a	Indicate the study’s design with a commonly used term in the title or the abstract	Title page
Abstract	#1b	Provide in the abstract an informative and balanced summary	Page i

		of what was done and what was found	
**Introduction**			
Background / rationale	#2	Explain the scientific background and rationale for the investigation being reported	Pages 1&2
Objectives	#3	State specific objectives, including any prespecified hypotheses	Page 2
**Methods**			
Study design	#4	Present key elements of study design early in the paper	Page 2
Setting	#5	Describe the setting, locations, and relevant dates, including periods of recruitment, exposure, follow-up, and data collection	Page 2 & 3
Eligibility criteria	#6a	Give the eligibility criteria, and the sources and methods of selection of participants.	Pages 3 & 4
	#7	Clearly define all outcomes, exposures, predictors, potential confounders, and effect modifiers. Give diagnostic criteria, if applicable	Page 3
Data sources / measurement	#8	For each variable of interest give sources of data and details of methods of assessment (measurement). Describe comparability of assessment methods if there is more than one group. Give information separately for exposed and unexposed groups if applicable.	Pages 3, 4 & 5
Bias	#9	Describe any efforts to address potential sources of bias	Page

For peer review only

			3,4,5&6
Study size	#10	Explain how the study size was arrived at	Pages 3&4
Quantitative variables	#11	Explain how quantitative variables were handled in the analyses. If applicable, describe which groupings were chosen, and why	Pages 5, 6, 10,11 & 12.
Statistical methods	#12a	Describe all statistical methods, including those used to control for confounding	Pages 5 & 6
Statistical methods	#12b	Describe any methods used to examine subgroups and interactions	Pages 5, 6, 9, 10 & 11
Statistical methods	#12c	Explain how missing data were addressed	Page 5
Statistical methods	#12d	If applicable, describe analytical methods taking account of sampling strategy	Pages 5& 6
Statistical methods	#12e	Describe any sensitivity analyses	Pages 5, 6, 9, 10&11

Results

Participants	#13a	Report numbers of individuals at each stage of study—eg numbers potentially eligible, examined for eligibility, confirmed eligible, included in the study, completing follow-up, and analysed. Give information separately for for exposed	Page 7

For peer review only

		and unexposed groups if applicable.	
Participants	#13b	Give reasons for non-participation at each stage	Page 7
Participants	#13c	Consider use of a flow diagram	n/a
Descriptive data	#14a	Give characteristics of study participants (eg demographic, clinical, social) and information on exposures and potential confounders. Give information separately for exposed and unexposed groups if applicable.	Page 7
Descriptive data	#14b	Indicate number of participants with missing data for each variable of interest	n/a
Outcome data	#15	Report numbers of outcome events or summary measures. Give information separately for exposed and unexposed groups if applicable.	Page 7 & 8
Main results	#16a	Give unadjusted estimates and, if applicable, confounder-adjusted estimates and their precision (eg, 95% confidence interval). Make clear which confounders were adjusted for and why they were included	Pages 9 to 11
Main results	#16b	Report category boundaries when continuous variables were categorized	Pages 9 to 11
Main results	#16c	If relevant, consider translating estimates of relative risk into absolute risk for a meaningful time period	n/a
Other analyses	#17	Report other analyses done—e.g., analyses of subgroups and interactions, and sensitivity analyses	Pages 9 to 11

For peer review only

Discussion

Key results	#18	Summarise key results with reference to study objectives	Pages 11 to 13
Limitations	#19	Discuss limitations of the study, taking into account sources of potential bias or imprecision. Discuss both direction and magnitude of any potential bias.	Page ii and 13 & 14
Interpretation	#20	Give a cautious overall interpretation considering objectives, limitations, multiplicity of analyses, results from similar studies, and other relevant evidence.	Page 14
Generalisability	#21	Discuss the generalisability (external validity) of the study results	Pages ii, 13 & 14

Other Information

Funding	#22	Give the source of funding and the role of the funders for the present study and, if applicable, for the original study on which the present article is based	Page ii

None The STROBE checklist is distributed under the terms of the Creative Commons Attribution License CC-BY. This checklist can be completed online using https://www.goodreports.org/, a tool made by the EQUATOR Network in collaboration with Penelope.ai
